# Is check-up on demand non-inferior to routine follow-up at one year after total hip or knee arthroplasty in terms of clinical outcomes and cost-effectiveness? Protocol for a randomized stepped-wedge hybrid effectiveness de-implementation trial

**DOI:** 10.1371/journal.pone.0343627

**Published:** 2026-03-17

**Authors:** Ariena J. Rasker, Lidy A. C. Roubos, Dominique C. Baas, Ronald A. W. Verhagen, Lex D. de Jong, Marijn Rutgers, Sigrid N. W. Vorrink, Jantsje H. Pasma, Rudolf W. Poolman, Nienke W. Willigenburg

**Affiliations:** 1 Department of Orthopaedic Surgery, Joint Research, OLVG Hospital, Amsterdam, the Netherlands; 2 Department of Orthopaedic Surgery, Leiden University Medical Center, Leiden, the Netherlands; 3 Department of Orthopaedic Surgery, Tergooi Medical Center, Hilversum, the Netherlands; 4 Department of Orthopaedics, Rijnstate Hospital, Arnhem, the Netherlands; 5 Department Orthopaedic Surgery, Reinier Haga Orthopedic Center, Zoetermeer, the Netherlands; PLOS: Public Library of Science, UNITED STATES OF AMERICA

## Abstract

**Background:**

Total hip arthroplasty (THA) and total knee arthroplasty (TKA) are highly effective surgical procedures for patients with end-stage osteoarthritis. Due to population ageing and the rising prevalence of osteoarthritis, the demand for these procedures continues to increase, placing pressure on healthcare systems. Postoperative follow-up care contributes to this burden, yet internationally its timing and frequency after THA and TKA differ substantially. Dutch guidelines recommend routine follow-up (RFU) at 6–12 weeks and 1 year postoperatively. However, most complications are identified based on symptoms, often during unplanned visits. Consequently, the added value of a 1-year routine follow-up visit remains unclear, suggesting that alternative follow-up strategies, such as check-up on demand (COD) might reduce unnecessary visits.

**Materials and methods:**

This multicenter hybrid type II effectiveness de-implementation trial uses a stepped-wedge cluster randomized design across 10 Dutch hospitals. All hospitals will sequentially transition from RFU with scheduled follow-up visits at 6–12 weeks and 1 year postoperatively to Check-Up on Demand (COD), in which patients have a scheduled visit at 6–12 weeks and receive a leaflet with instructions on when and how to contact the hospital, without a scheduled 1-year visit. A total of 1,000 patients aged ≥50 years undergoing primary THA or TKA for osteoarthritis will be included. Each participating hospital will recruit 100 patients (50 THA and 50 TKA). The primary clinical outcome is PROMIS physical functioning at 2 years (i.e., 1 year after the 1-year follow-up moment). The primary process outcome is the number of patients who have a clinical visit or X-ray related to surgery at 1 year postoperatively. Secondary outcomes include complications, surgical interventions, additional healthcare consumption, quality of life, pain, satisfaction, and costs. An economic evaluation and budget impact analysis will be conducted from healthcare and societal perspectives. The trial is registered at ClinicalTrials.gov (NCT06971757).

## Introduction

Total Hip Arthroplasty (THA) and Total Knee Arthroplasty (TKA) are two of the most common surgical procedures for patients suffering from end-stage osteoarthritis. These surgeries are highly effective in reducing pain and improving mobility [1,2]. In 2021, high-income countries reported average rates of 172 hip arthroplasties and 119 knee arthroplasties per 100,000 individuals, which is equivalent to more than 2.2 million hip and 1.5 million knee procedures annually [3]. Given the ageing population and the rising prevalence of osteoarthritis, the pressure on the healthcare system is increasing [4,5]. Follow-up care contributes substantially to this burden, both in terms clinical workload and costs. It is unknown whether routine follow-up (RFU) visits actually improve patient outcomes, for example by earlier detection of complications or by supporting patients in regaining confidence and physical function, an essential patient-reported outcome after THA or TKA [6,7]. Alternative strategies, such as check-up on demand (COD) are therefore being considered.

The timing and frequency of follow-up care after THA and TKA differs substantially between countries [8–10]. Local policies and surgeon perferences often drive this variation, as solid evidence is lacking. In the Netherlands, a nationwide survey conducted in 2019 among 111 surgeons revealed substantial practice variation, with the number of routinely scheduled follow-up visits ranging from 3 to 7 per patient within a follow-up period of 15 years (S2 File). In addition, a nationwide patient survey conducted between 2021 and 2023 among 403 THA and TKA patients showed that most patients preferred follow-up on demand and indicated that they would contact their healthcare provider themselves in case of symptoms (S3 File).

Dutch national guidelines recommend routine follow-up at 6–12 weeks after surgery to assess wound healing and early recovery, followed by a clinical visit including a radiograph (X-ray) at 1 year. To assess implant survival and detect complications on the longer term, continued follow-up is advised at or beyond 5 years postoperatively for THA, and may be considered every 5 years for TKA [11,12]. While the early postoperative visit at 6–12 weeks is widely accepted based on clinical rationale, recommendations for subsequent routine follow-up are not evidence-based, as they rely on very low-quality evidence.

Registry data show that revisions in the first year occur in approximately 2% of THA patients and around 1% of TKA patients [13,14]. A prospective cohort study, supported by a narrative review, showed that revisions are predominantly preceded by the onset of symptoms, with over 95% of revision procedures being performed for symptomatic indications, thereby questioning the value of routine follow-up [15,16]. Consistently, more than 90% of routine follow-up visits beyond the first postoperative year did not result in a change in patient management [17], and it is typically the patients with symptoms that present for follow-up [18,19]. Building on this evidence, Kingsbury et al. (2022) concluded that it is safe to disinvest from routine follow-up between 1 and 10 years after uncomplicated THA and TKA [20]. However, this report did not specifically assess follow-up at 1 year, nor did it evaluate outcomes such as clinical effectiveness, number of follow-up visits, or cost-effectiveness. Nevertheless, a 1-year routine follow-up visit was still consistently scheduled by the majority of orthopedic surgeons (85.4%) according to the nationwide surgeon survey (S2 File), indicating that the results of its evaluation has a large potential impact on healthcare efficiency.

The primary objective of this study is to evaluate the differences in physical functioning and number of patients with a clinical visit X-ray between check-up on demand (COD) and routine follow-up (RFU) at 1 year after THA or TKA. Secondary outcomes include complications, surgical interventions, additional healthcare consumption, quality of life, pain, and costs. This study will generate high quality and directly applicable evidence to inform clinical guidelines regarding THA and TKA follow-up care.

## Methods and materials

### Design

**Fig 1:**
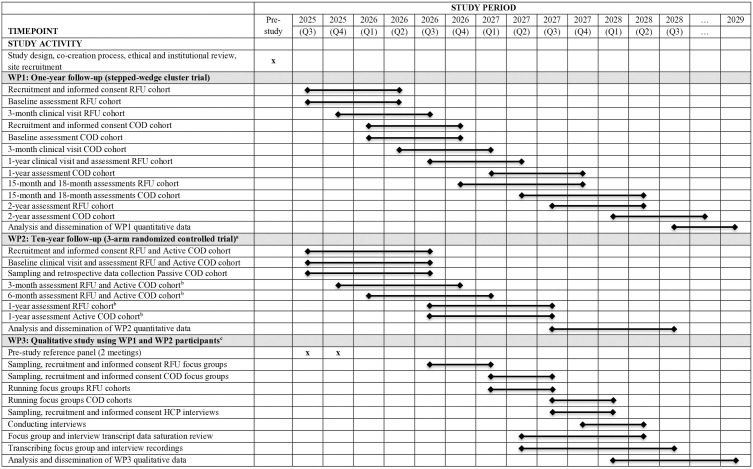
SPIRIT Schedule of Enrollment, Interventions, and Assessments summarizing all 3 work packages of the nationwide hybrid effectiveness de-implementation trial (adapted to fit the study design). a This part of the study recruits patients who underwent total hip or knee arthroplasty approximately 10 years ago. Only the RFU and Active COD cohorts will undergo prospective assessments, while for the Passive COD cohort only retrospective registry data will be collected. b The time points referred to as “3-month,” “6-month,” and “1-year” assessments should be understood as 10 years + 3 months, 10 years + 6 months, and 11 years after surgery. These labels reflect the time elapsed since enrolment in this follow-up phase, not since the original procedure. c This part of the study is a nested qualitative study including focus groups with patients from WP1 and WP2 and interviews with health care professionals. It explores experiences, perceptions, and acceptability of RFU and COD in order to identify barriers and facilitators for implementation. This SPIRIT schedule is adapted from the figure published in 2025 in the related qualitative study by de Jong et al. [22]. COD, check-up on demand; HCP, health care professional; RFU, routine follow-up; WP, Work Package.

This study is designed as a hybrid effectiveness de-implementation trial type II, evaluating both clinical effectiveness and implementation outcomes [21]. It is part of a nationwide project with three work packages (WPs): WP1 (current study) evaluates the 1-year follow-up, WP2 will assess the 10-year follow-up, and WP3 is a qualitative study on patient and healthcare professional HCP experiences with RFU and COD [22]. The SPIRIT Schedule of Enrollment, Interventions, and Assessments (Fig 1) summarizes all work packages within the project. The project is embedded within the Dutch *Zorgevaluatie & Gepast Gebruik* (ZE&GG) program, which aims to promote evidence-based and appropriate use of healthcare through nationwide evaluation and implementation projects (https://www.zorgevaluatienederland.nl/evaluations/haka-1-year-fu).

### Ethics and registration

The study was registered on 13 May 2025 at ClinicalTrials.gov (ID: NCT06971757, https://clinicaltrials.gov/study/NCT06971757). The study protocol (version 2.1, dated 15 May 2025) was approved by the Medical Research Ethics Committee of Leiden–Den Haag–Delft (METC LDD) (reference number: NL-009246) on 19 May 2025, and by the local ethics boards of all 10 participating hospitals. The approved study protocol is added as S4 File. Any important protocol modifications will be submitted to the Medical Research Ethics Committee for approval and communicated to relevant parties, including local ethics boards, investigators, and the trial registry.

### Stakeholder and patient involvement

The study design was co-created with stakeholders within the framework of the Dutch ZE&GG program. This process involved input from patients, researchers, healthcare professionals, as well as stakeholders from various disciplines, including general practitioners, physiotherapists, epidemiologists, health economists, health insurers, guideline developers, implementation experts, representatives of the Dutch healthcare institute, and members of the Dutch Orthopaedic Association (*Nederlandse Orthopaedische Vereniging, NOV*). Through a series of structured co-creation meetings, held between July 2023 and February 2024, the perspectives and expertise of these stakeholders were integrated into the design of the study, addressing both methodological and practical aspects, such as feasibility, outcome selection, sample size, and implementation strategies. Their continued involvement will guide the project, and all relevant stakeholders involved in the co-creation process have formally endorsed the study, confirming its relevance and methodological quality, and supporting the implementation of its results in clinical practice.

Two patient representatives, affiliated with the Poly-Artrose Lotgenoten Vereniging (Dutch Polyarthritis Patient Association) and Reumazorg Nederland (Dutch Rheumatology Patient Organization), are actively involved in the project. They contributed to the co-creation process and continue to advise across all phases. Their involvement is documented in a participation matrix outlining contributions ranging from advisory roles to shared decision-making responsibilities (S5 File).

### Participants

Participants will be recruited at 10 non-academic hospitals across the Netherlands. Inclusion criteria are: scheduled for primary THA or TKA surgery; a painful and disabled hip or knee joint caused by osteoarthritis; age 50 years or older at the time of surgery; capable and willing to complete questionnaires; proficiency in the Dutch or English language; and willingness to provide informed consent. Exclusion criteria are: an indication for surgery other than osteoarthritis; scheduled for revision arthroplasty (except conversion from unicompartmental knee arthroplasty to TKA or from hip hemiarthroplasty/resurfacing to THA); scheduled for bilateral THA or TKA; or prior participation in this study due to earlier hip or knee arthroplasty.

### Recruitment and consent

Eligible patients on the waiting list or scheduled for THA or TKA will be invited to participate in the study prior to surgery. Participant enrollment and the informed consent procedure will be carried out by trained physicians or researchers at each participating hospital. Depending on the patient’s preference, informed consent can be provided either digitally or in writing. Digital consent is obtained via Castor EDC (Castor, Amsterdam, The Netherlands), a certified electronic data capture system compliant with data protection and security standards. Patients are informed that participation is voluntary and that they may withdraw from the study at any time without consequences for their medical care.

### Randomization and allocation

This study uses a stepped-wedge cluster trial design in which 10 hospitals will sequentially transition from routine follow-up (RFU) to check-up on demand (COD). The order of transition of the participating hospitals was determined by cluster randomization, performed by the initiating hospital (OLVG) on 20 May 2025 using R version 4.2.2 with RStudio 2024.04.2 (R Foundation for Statistical Computing, Vienna, Austria). A computer-generated randomization sequence was created using a pre-specified random seed to ensure reproducibility. All 10 participating hospitals were randomly ordered and subsequently divided into 5 groups of 2 hospitals each, determining their stepwise transition order.

Each hospital will recruit patients scheduled for THA and TKA, starting with 2 patients per procedure per month and gradually increasing to 5 patients per procedure per month (Table 1). Over a 14-month inclusion period, each site is expected to recruit 100 patients, yielding a total sample size of 1,000. This design supports a realistic, phased de-implementation of RFU. A two-month wash-out period is planned to facilitate proper de-implementation of RFU and implementation of COD. During this transition period, protocols, patient information materials, automated follow-up systems, and communication workflows will be updated, relevant healthcare professionals will be informed, and evaluation activities will be integrated.

**Table 1:** Anticipated monthly patient inclusion per hospital in the stepped-wedge design.

Period	Center 1	Center 2	Center 3	Center 4	Center 5	Center 6	Center 7	Center 8	Center 9	Center 10	Total	
	Anticipated included patients*											Cumulative
jul-25	4	4	4	4	4	4	4	4	4	4	40	40
aug-25	6	6	6	6	6	6	6	6	6	6	60	100
sep-25	10	10	10	10	10	10	10	10	10	10	100	200
oct-25	10	10	10	10	10	10	10	10	10	10	100	300
nov-25	Transition	Transition	10	10	10	10	10	10	10	10	80	380
dec-25	Transition	Transition	Transition	Transition	10	10	10	10	10	10	60	440
jan-26	10	10	Transition	Transition	Transition	Transition	10	10	10	10	60	500
feb-26	10	10	10	10	Transition	Transition	Transition	Transition	10	10	60	560
mar-26	10	10	10	10	10	10	Transition	Transition	Transition	Transition	60	620
apr-26	10	10	10	10	10	10	10	10	Transition	Transition	80	700
may-26	10	10	10	10	10	10	10	10	10	10	100	800
jun-26	10	10	10	10	10	10	10	10	10	10	100	900
jul-26	6	6	6	6	6	6	6	6	6	6	60	960
aug-26	4	4	4	4	4	4	4	4	4	4	40	1000
Total	100	100	100	100	100	100	100	100	100	100	1000	
Routine follow-up (RFU)												
Check-up on demand (COD)												

*Numbers represent inclusion of patients scheduled for THA or TKA, starting with 2 patients per month for each surgery, followed by 3 patients per month, and then 5 patients scheduled for THA and 5 patients scheduled for TKA per month.

### Intervention (Check-up On Demand, COD)

All patients receive postoperative follow-up within 3 months after surgery as part of standard care, typically at 6–12 weeks, either in-person or by telephone, depending on local hospital practice. During this visit, patients in the COD group will receive a written information leaflet describing when and how to contact the hospital in case of pain, concerns, or symptoms related to THA or TKA. This leaflet, developed with input from patient representatives and surgeons, is included in S6 File. No routine follow-up visit at 1 year will be scheduled; any further check-ups may be initiated on demand by the patient or healthcare professional in response to symptoms, concerns, or uncertainty regarding recovery.

### Comparison (Routine Follow-up, RFU)

The comparator group will receive RFU, in accordance with current Dutch guidelines. Similar to the intervention group, all patients receive postoperative follow-up within 3 months after surgery as part of standard care, typically at 6–12 weeks, either in-person or by telephone, depending on local hospital practice. In addition, RFU includes a standardized 1-year follow-up consisting of a clinical visit and X-ray. As in COD, patients may initiate a check-up on demand any time, in addition to their scheduled 1-year visit.

### Crossover

Crossover between intervention and comparison group is not applicable, as no formal criteria for discontinuing or modifying the allocated interventions apply. Participants who do not attend their scheduled RFU visit will remain in the RFU group and their outcomes will be measured according to the protocol. Non-attendance will be recorded and analyzed as part of the process outcomes, as it provides relevant information on the adherence to routine follow-up. Similarly, patients who are in the COD group and request a 1-year follow-up are not considered crossovers; these patients just make use of the opportunity for a check up on demand.

### Outcomes

Participants will be followed until 2 years after surgery, which corresponds to 1 year after receiving either RFU or COD. During the co-creation meeting, patient representatives identified physical function as the most important outcome after THA or TKA. As a result, physical functioning was selected as the primary clinical outcome. Additionally, a process outcome was defined to assess the de-implementation of RFU and evaluate the effectiveness of the new follow-up strategy. The outcomes are defined as follows:

- *Clinical:* Physical functioning, measured with the Patient-Reported Outcomes Measurement Information System (PROMIS^®^) Physical Function Short Form 10b v2.0 [23]. The short form consists of 10 items on daily activities and yields a raw sum score (10–50) that can be converted to a standardized T-score (mean 50, SD 10; higher scores indicate better functioning). Measurement properties of PROMIS short forms have demonstrated high reliability, good precision, low burden, and minimal floor/ceiling effects in patients after THA [24,25]. PROMIS physical function will be assessed at baseline (before surgery), and 12, 15, 18 and 24 months after surgery, with the primary endpoint defined as physical functioning at 24 months.
- *Process:* The number of patients who have a clinical visit or X-ray related to the surgery at 12 months (plus or minus 60 days) postoperatively, recorded in the electronic health records system of the hospital where the primary surgery was performed.

Secondary outcomes include:

- Number and type of complications. These include infection, periprosthetic fracture, loosening, malalignment or malposition of components, prosthetic wear, dislocation, and any other complications related to THA or TKA recorded during follow-up.
- Number and type of surgical interventions. These include debridement, antibiotics and implant retention (DAIR), irrigation and debridement with component exchange, partial component revision, full revision surgery, and any other procedures related to THA or TKA recorded during follow-up.
- Additional healthcare consumption in the hospital related to THA or TKA: number of clinical visits, telephone consultations, X-rays, CT scans, MRI scans, laboratory tests, and any other types of care recorded during follow-up.
- Additional healthcare consumption outside the hospital related to THA or TKA: visits to the general practitioner (in-person visits, telephone consultations, or home visits), therapists (e.g., physiotherapists, occupational therapists), and home care services. These data will be obtained through a study-specific resource use and cost questionnaire to capture potential shifts in healthcare utilization (e.g., patients in the COD group not attending a 1-year hospital visit but consulting their general practitioner multiple times instead).
- Health-related quality of life, measured by the EQ-5D-5L, using validated Dutch and English versions [26]. The instrument assesses five dimensions (mobility, self-care, usual activities of daily living, pain/discomfort, anxiety/depression), each with five response levels, and includes a visual analogue scale (VAS) from 0–100 for overall health. Responses can be converted into an index score using country-specific value sets [27]. Developed by the EuroQol Group, the EQ-5D-5L has shown good construct validity, acceptable test–retest reliability, and responsiveness in THA populations [28–30]. The EQ-5D-5L will be assessed at baseline (before surgery), 12, 15, 18 and 24 months after surgery.
- Pain, measured with the Numeric Pain Rating Scale (NPRS). Patients are asked to rate pain intensity related to THA or TKA during the past week, both at rest and during activity, on a scale from 0 (no pain) to 10 (worst possible pain). Pain will be assessed at baseline (before surgery), 12, and 24 months after surgery.
- Satisfaction, measured with the Numeric Satisfaction Rating Scale (NSRS). Patients are asked to rate their overall satisfaction with the outcome of their THA or TKA on a scale from 0 (very dissatisfied) to 10 (very satisfied). Satisfaction will be assessed at 12, and 24 months after surgery.
- Healthcare costs, derived from the study-specific resource use and cost questionnaire, which was also used to assess healthcare consumption. This covers a wide range of resource use including visits to the general practitioner, physical therapist, rehabilitation center, nursing home, other hospitals than the one where the surgery was performed), use of assistive devices and medications, formal and informal care (including care at home by professionals or proxy’s), and productivity losses (including absenteeism and presenteeism at a paid job, as well as unpaid productivity such as volunteer work and care for others) related to the THA or TKA. Costs will be assessed at 15, 18 and 24 months after surgery.

The following characteristics will be recorded:

- *Patient characteristics*: age, gender, Body Mass Index (BMI), American Society of Anesthesiologists (ASA) score, ethnicity, employment status, and education level.
- *Surgical characteristics*: laterality, surgical approach (direct lateral, posterolateral, anterolateral, anterior, or direct superior for THA; and medial parapatellar, lateral parapatellar, midvastus, or subvastus for TKA), fixation method (cemented, uncemented, or hybrid), surgical duration, and intra-operative complications (e.g., excessive bleeding, neurovascular injury, fracture, or damage to ligaments or soft tissues).
- *Postoperative characteristics*: length of hospital stay, in-hospital complications (e.g., deep venous thrombosis, pulmonary embolism, joint infection, persistent wound leakage, prosthesis dislocation, delirium, urinary tract infection, or anemia requiring transfusion), and discharge destination (home without assistance, home with activities of daily living support, nursing home, rehabilitation center, or other).

### Data collection and management

Study data will be collected in a secure electronic Case Report Form (eCRF) within Castor Electronic Data Capture (EDC). Castor EDC provides validated data entry with role-based access, audit trails for all data changes, and automated back-ups. Questionnaires can be completed online via Castor EDC or on paper and require approximately 10–25 minutes per time point. A maximum window of ±30 days will be applied for data collection of the questionnaires. Clinical and administrative data will be extracted from electronic health records and entered into the eCRF manually, following a detailed standard operating procedure. An overview of study procedures and timing of all assessments is provided in Table 2.

**Table 2:** Overview of the study procedures and timing of assessments.

	Before surgery	Surgery	Discharge	3 months	1 year: RFU or COD	15 months	18 months	24 months
Informed consent procedure	**✓**							
Demographic and clinical								
Patient characteristics	**✓**							
Surgical characteristics		**✓**						
Postoperative characteristics			**✓**					
Intervention								
Clinical visit				**✓**	**✓** (for RFU)			
X-ray				**✓**	**✓** (for RFU)			
Outcomes								
Physical function	**✓**				**✓**	**✓**	**✓**	**✓**
Clinical visit or X-ray								**✓**
Complications								**✓**
Surgical interventions								**✓**
Healthcare consumption								**✓**
Quality of life	**✓**				**✓**	**✓**	**✓**	**✓**
Pain	**✓**				**✓**			**✓**
Satisfaction					**✓**			**✓**
Healthcare costs						**✓**	**✓**	**✓**

RFU, routine follow-up; COD, check-up on demand.

Data management and storage procedures are described in a study-specific Data Management Plan (S7 File). Personal information about participants will be coded to protect their identities, using a subject identification code list that is stored securely and separately from the research data at each site. Access to personal data is restricted to authorized study personnel. Data will be handled in accordance with the Dutch General Data Protection Regulation (GDPR) and other relevant national legislations.

Data will be stored on secure servers with restricted access and retained for at least 15 years. Monitoring will be performed in all participating hospitals according to the study-specific monitoring plan, in line with Good Clinical Practice guidelines. This includes on-site and remote monitoring, verification of informed consent procedures, and source data verification of a sample of participants. The Medical Research Ethics Committee granted a waiver for the requirement to establish a Data Steering and Monitoring Committee, as no additional risks are expected and both RFU and COD are part of standard care.

### Safety considerations

Only adverse events (AEs) and serious adverse events (SAEs) directly related to the presence or absence of the 1-year clinical visit and X-ray will be reported. All other complications, surgical interventions, and healthcare consumption related to the THA or TKA are systematically collected as part of the outcome measures, but not reported as (S)AEs.

In accordance with Section 10, Subsection 4 of the Dutch Medical Research Involving Human Subjects Act (WMO), the sponsor will suspend the study if there is sufficient ground that continuation would compromise participant health or safety. The Medical Research Ethics Committee will be notified without undue delay, and participants will be informed accordingly.

### Sample size

The sample size was determined in collaboration with the co-creating stakeholders. To define what would be considered convincing evidence for de-implementation in daily practice, a survey was distributed to members of the NOV. Twenty-five respondents completed the survey, including orthopaedic surgeons, residents, researchers, and physician assistants. They identified the clinically relevant threshold on the PROMIS physical function scale and the required number of patients per intervention group required to impact their clinical practice. Nearly 90% of respondents considered a difference of at least 5 points on the primary outcome (PROMIS physical functioning) to be clinically relevant.

Although the minimal clinically important difference (MCID) for PROMIS physical function varies by version, population, and methodology [31], published estimates generally range between 3 and 5 points. For example, Humphrey et al. reported an MCID of 3.27 for the PROMIS physical function short form [32]. In the context of a non-inferiority design, a margin smaller than the difference considered clinically relevant by stakeholders was therefore deliberately chosen, resulting in a conservative non-inferiority margin of 3 points. Accordingly, a sample size calculation was performed using the Sealed Envelope online tool [33]. Assuming a standard deviation of 9 for the PROMIS physical function score [25] and a non-inferiority margin of 3 points, a total of 190 patients per group would be required to achieve 90% power with a one-sided 97.5% confidence interval. Anticipating 20–25% loss to follow-up, a sample size of 250 patients per group (500 THA and 500 TKA patients in total) is expected to provide sufficient power for the primary analysis. More importantly, as power and sample size calculations are highly dependent on assumptions that cannot be verified a priori, a total sample size of 500 THA and 500 TKA was considered to provide sufficient evidence to adopt the trial’s results in clinical practice by 96% of the respondents to our survey.

### Statistical analyses

All analyses will be conducted according to both the intention-to-treat (ITT) and per-protocol (PP) principles and will be performed separately for the THA and TKA patient groups. In line with recommendations for the reporting and interpretation of non-inferiority trials, both ITT and PP analyses will be performed to evaluate the primary outcomes, with the PP analysis serving as a complementary, conservative analysis [34]. Statistical analyses will be conducted using SPSS Statistics version 29.0 (IBM Corp., Armonk, NY, USA) and R version 4.5.2 with RStudio 2025.09.2−148 (R Foundation for Statistical Computing, Vienna, Austria), or newer versions if available at the time of analysis.

Categorical variables will be summarized as frequencies and percentages. Continuous variables will be summarized as means with standard deviations for normally distributed data and as medians with interquartile ranges for non-normally distributed data; ordinal outcomes such as satisfaction scores will be summarized as medians with interquartile ranges.

Missing data will be minimized by contacting participants in cases of incomplete questionnaire responses. For outcomes extracted from the medical records, we expect very few missing values. For the primary and secondary longitudinal outcomes, mixed effects models and generalized estimating equations (GEE) can handle missing values under the assumption that data are missing (completely) at random. After database closure, the extent and patterns of missingness will be examined. If substantial missing data are observed, multiple imputation using chained equations will be conducted as a sensitivity analysis to assess the robustness of the results [35,36]. For all primary and secondary outcomes, analyses will be performed both unadjusted and adjusted for potential confounders, to assess the robustness of the findings.

#### Primary outcomes.

The primary analysis will evaluate non-inferiority of COD compared with RFU at 2 years postoperatively, using a non-inferiority margin of 3 points on the PROMIS physical functioning score. The estimated mean difference between groups at 2 years will be obtained from a linear mixed model (LMM). Non-inferiority will be concluded when the upper bound of the 95% confidence interval does not exceed 3 points.

Differences in PROMIS physical function between the RFU and COD groups at different time points (12, 15, 18 and 24) will be compared using LMM. Repeated measurements are clustered within participants, and participants clustered within hospitals using nested random intercepts. Fixed effects are intervention (RFU vs. COD), time (12, 15, 18 and 24), baseline PROMIS physical function, and the interaction of intervention*time to determine the crude effect of the intervention at each time point. Given the stepped-wedge design, the timing of the transition to the intervention differs between sites. Therefore, variable “step” will also be included as a categorical fixed effect. Results will be presented with marginal mean differences and 95% confidence intervals (CI).

The number of patients who have had at a clinical visit or X-ray 1 year after RFU or COD will be presented for each group. GEE with a binomial link function will be used to compare the presence or absence of visits/X-rays (yes/no per patient) between RFU and COD. Measurements are clustered for hospital and models include fixed effects for intervention and step. Results are presented as odds ratio and 95% CI.

#### Secondary outcomes.

For complications, surgical interventions, and additional healthcare consumption inside and outside the hospital, the percentage of patients who have experienced each will be presented for each group. GEE with a binomial function will be used to compare the presence or absence of these outcomes (yes/no per patient) between RFU and COD. Additionally, the total number of complications, interventions, and additional healthcare consumption inside and outside the hospital per patient will be analyzed using GEE with a negative binomial function. The GEE models will be built similarly to the model for the number of patients who have had a clinical visit or X-ray, with results presented as rate ratios (RR). The type of complication, intervention, and additional healthcare consumption inside and outside the hospital will be analyzed descriptively at each time point. Health-related quality of life (EQ-5D-5L), pain (NPRS), and satisfaction (NSRS) will be analyzed using LMMs with a similar structure as the model for the PROMIS physical function.

### Cost-effectiveness

The trial-based economic evaluation will focus on the primary clinical outcome (PROMIS physical function) and QALYs. Analyses will be conducted from both the societal and healthcare perspectives. QALYs will be calculated by multiplying utility values derived from the EQ-5D-5L (valued using the Dutch tariff) by time spent in each health state [27,37]. Resource use will be measured at 15, 18, and 24 months via a study-specific questionnaire and valued according to the Dutch costing manual [38]. Societal costs will include healthcare utilization, use of assistive devices and medications, formal and informal care, and productivity losses (absenteeism, presenteeism, and unpaid productivity); healthcare costs will cover formal care within the Dutch system.

LMM will be used to estimate cost and effect differences, with repeated measures clustered within participants and participants clustered within hospitals (random intercepts). Incremental cost-effectiveness ratios will be calculated as cost differences divided by effect differences, with uncertainty assessed through non-parametric bootstrapping nested within multiple imputation. Results will be visualized in cost-effectiveness planes and acceptability curves [39,40]. Sensitivity analyses, including complete-case analysis, will test result robustness [37].

A budget impact analysis (BIA) will be conducted using ZonMw’s BIA tool, based on Dutch incidence data and perspectives of society, government (Budget Kader Zorg), and health insurers. Scenarios will include: (1) continued RFU for all, (2) COD for all, and (3) COD for selected low-risk subgroups, defined by trial results. Cost estimates will use Dutch standard prices, tariffs set by the Dutch Healthcare Authority (NZA), or average insurer tariffs. The cost-effectiveness and BIA analyses will adhere to the ‘Dutch guideline for economic evaluations in healthcare’ [41].

### Study status and timeline

Participant recruitment started in July 2025 (first participant enrolled on 1 July 2025) and is expected to be completed in August 2026. Data collection will be completed in August 2028, followed by analysis and final reporting by December 2029.

## Discussion

Follow-up care after THA and TKA represents a substantial and growing part of orthopaedic healthcare, yet the optimal timing and frequency of RFU remain unclear. Despite limited evidence supporting its added clinical value, a routine clinical visit with radiographic assessment at 1 year postoperatively continues to be standard practice in the Netherlands. Given that most complications after THA and TKA are typically identified following the onset of symptoms, robust evidence is needed to determine whether this routine visit can be safely and efficiently replaced by alternative follow-up strategies.

This multicenter stepped-wedge cluster randomized hybrid effectiveness de-implementation trial will evaluate whether RFU at 1 year after THA or TKA can be safely and cost-effectively replaced by COD. This study has several notable strengths. First, the stepped-wedge design allows all participating hospitals to contribute data across both approaches facilitating a pragmatic and gradual transition to de-implementation of RFU. Second, the hybrid type II design ensures that both clinical effectiveness and implementation aspects are addressed, enhancing its relevance for guideline developers and healthcare policy. Third, the study was co-created with a broad group of stakeholders and patient representatives, which strengthens the clinical applicability and potential for national implementation. Finally, the inclusion of an economic evaluation and a BIA is crucial given the growing demand for THA and TKA and the increasing pressure on healthcare resources.

Some limitations should also be considered. While the stepped-wedge design is well suited for pragmatic de-implementation trials, time effects and learning effects may introduce bias. However, these factors will be addressed in the statistical analyses and are anticipated to be minimal, given the relatively short enrollment period of approximately 14 months. Moreover, the current (pre-study) clinical practice differs between hospitals, i.e., some have already implemented COD but will temporarily switch back to RFU to comply to the study design. In addition, in de-implementation trials there is a risk of selective adherence to the intervention, for example if clinicians feel reluctant to apply COD to all eligible patients and continue to schedule follow-up visits based on individual clinical judgement. Another limitation is that patient participation requires completion of questionnaires, which may pose challenges for individuals with limited digital or health literacy and introduce potential selection bias. To minimize this risk, questionnaires are available in both Dutch and English and can be completed either digitally or on paper.

In conclusion, this study will be the first to comprehensively investigate the 1-year follow-up visit after THA and TKA, explicitly incorporating clinical and process outcomes alongside healthcare utilization and costs. Given that the 1-year visit is currently the most consistently scheduled routine visit, the findings of this trial have the potential to substantially reduce unnecessary care and optimize the use of healthcare resources, without compromising patient outcomes. Stakeholders provided prior commitment to incorporate these findings into clinical practice and guideline development.

### Dissemination of findings

The findings of this study will be disseminated to participating hospitals, healthcare professionals, and relevant stakeholders through peer-reviewed publications, presentations at national and international conferences, and reporting in the ClinicalTrials.gov results database. There are no restrictions on publication. Authorship will adhere to the International Committee of Medical Journal Editors (ICMJE) criteria. A lay summary of the main findings will be provided to study participants and patient organizations involved in the study. The results will also be shared with the Dutch Orthopaedic Association and stakeholders within the ZE&GG program to inform guideline development and support implementation in clinical practice.

## Supporting information

S1 File SPIRIT checklist. (PDF)

S2 File Survey on current follow-up practices among surgeons. Conference abstract.
(PDF)

S3 File Survey on follow-up preferences of THA and TKA patients. Conference abstract.
(PDF)

S4 File Study protocol approved by the ethics committee. (PDF)

S5 File Patient involvement participation matrix. (PDF)

S6 File Information leaflet for check-up on demand (COD). (PDF)

S7 File Data management plan. (PDF)

S8 File Patient information and consent form. (PDF)

S9 File Postoperative follow-up questionnaire at 2 years after surgery. (PDF)
