# Supplementary material for: Is check-up on demand non-inferior to routine follow-up at one year after total hip or knee arthroplasty in terms of clinical outcomes and cost-effectiveness? Protocol for a randomized stepped-wedge hybrid effectiveness de-implementation trial
Source: PLoS One. 2026 Mar 17;21(3):e0343627. doi: 10.1371/journal.pone.0343627 (PMC12994803; doi:10.1371/journal.pone.0343627)
Supplement: S2 File — Conference abstract. (PDF) [file pone.0343627.s002.pdf]

Follow-up after total hip arthroplasty: results of a survey among orthopaedic surgeons in the Netherlands (NOV Congress, 7 October 2022).

J.H. Pasma, D.C. Baas, N.W. Willigenburg, R.W. Poolman, A.M.J.S. Vervest, M. Rutgers

Background and objective

After total hip arthroplasty (THA), patients are scheduled for follow-up visits to enable early detection of prosthesis failure and to prevent complex revision surgery associated with a high risk of complications and substantial costs. Current Dutch guidelines recommend follow-up at 1, 5, 10, and 15 years postoperatively. However, these recommendations are not based on high-quality evidence, resulting in patients not always attending their follow-up visits at these time points or patients have more follow-up visits than necessary. This study aims to describe current clinical practice and orthopaedic surgeons' views regarding follow-up after THA.

Methods

An online survey was distributed among members of the Dutch Orthopaedic Association (Nederlandse Orthopaedische Vereniging, NOV) via an internal message (16 October 2019), the newsletter (7 November 2019), and during the NOV autumn conference 2019. The survey comprised 26 questions on the frequency and content of current follow-up after THA and on perspectives regarding follow-up.

Results

A total of 111 orthopaedic surgeons completed the survey. Of these, 76.9% reported seeing patients three times or more after THA, with a maximum of seven visits; 48.7% indicated that they would prefer to see patients less frequently. The majority (85.4%) scheduled a follow-up visit at 1 year and 42.3% at 5 years. In most cases, the orthopaedic surgeon conducted the follow-up visit (87.0%), although 51.3% stated that a physician assistant could perform these visits. Almost all respondents (94.6% and 99.1%) reported adapting the follow-up schedule in the presence of radiographic abnormalities, both in asymptomatic and symptomatic patients.

Conclusion

There is substantial practice variation in follow-up after THA. High-quality randomized studies are needed to determine the optimal follow-up strategy after THA and to provide evidence-based recommendations to both patients and healthcare professionals.
