# Supplementary material for: Is check-up on demand non-inferior to routine follow-up at one year after total hip or knee arthroplasty in terms of clinical outcomes and cost-effectiveness? Protocol for a randomized stepped-wedge hybrid effectiveness de-implementation trial
Source: PLoS One. 2026 Mar 17;21(3):e0343627. doi: 10.1371/journal.pone.0343627 (PMC12994803; doi:10.1371/journal.pone.0343627)
Supplement: S3 File — Conference abstract. (PDF) [file pone.0343627.s003.pdf]

Follow-up after total hip and knee arthroplasty: insight in the needs of patients (NOV-NOF Congress, 12 June 2024).

J.H. Pasma, D.C. Baas, A.M.J.S. Vervest, M. Rutgers

Objectives

Guidelines recommend routine follow-up (RFU) after total hip and knee arthroplasty (THA and TKA) within 3 months, after 1 and after 5 years (THA) or every 5 years (TKA) to detect problems at an early stage. Considerable variation exists between Dutch hospitals and the evidence for RFU is low. This study provides insight in the perspectives and satisfaction of patients about follow-up.

Methods

A survey was sent from 12 hospitals to 440 THA and 440 TKA patients at different stages after surgery. The survey consisted of 38 questions about current follow-up and perspectives on follow-up. The survey was developed by an expert panel of 2 orthopedic surgeons and 2 researchers.

Results

The survey response rate was 45.8% (THA: 217; TKA: 186). 65.9% THA and 67.4% TKA patients were (very) satisfied with their current follow-up. Both RFU and X-rays were indicated as reasonably to extremely important (THA: 66.5-79.1%; TKA:74.0-81.0%). Patients preferred follow-up by an orthopedic surgeon (86.8-89.3%) mainly at 6 weeks (18.5-22.9%), 1 year (18.5-20.1%) and 5 years (13.4-16.1%) after surgery. Most patients (46.9% THA and 35.8% TKA patients) preferred follow-up on demand and will contact the hospital (69.0-72.6%) in case of complaints.

Conclusion

THA and TKA patients prefer follow-up on demand over routine follow-up. More evidence is needed to safely replace routine follow-up by follow-up on demand. Follow-up on demand requires clear instructions for patients when to contact which healthcare professional.
