# Supplementary material for: Is check-up on demand non-inferior to routine follow-up at one year after total hip or knee arthroplasty in terms of clinical outcomes and cost-effectiveness? Protocol for a randomized stepped-wedge hybrid effectiveness de-implementation trial
Source: PLoS One. 2026 Mar 17;21(3):e0343627. doi: 10.1371/journal.pone.0343627 (PMC12994803; doi:10.1371/journal.pone.0343627)
Supplement: S4 File — (PDF) [file pone.0343627.s004.pdf]

RESEARCH PROTOCOL

Routine follow-up (FU) at 1 year after Hip- And Knee Arthroplasty (HAKA): wasting resources or appropriate healthcare?

PROTOCOL TITLE '*Routine follow up at 1 year after Hip- And Knee Arthroplasty (HAKA): wasting resources or appropriate healthcare?*'

**Protocol ID**	**WO 24.0150**
**Short title**	**HAKA trial 1 year FU**
**Version**	**2.1**
**Date**	**15-05-2025**
**Project leader**	**Dr. N.W. Willigenburg**
**Coordinating investigator(s)**	**L.A.C. Roubos**
**Sites multicenter research**	**The participating centers and Principal Investigators are listed in the ABR form.**
**Sponsor**	**OLVG**
**Subsidising party**	**ZonMw**
**Independent expert(s)**	**Dr. Ing. Sebastiaan van de Groes**

PROTOCOL SIGNATURE SHEET

Name	Signature	Date
Head of Department:<br>Dr. E.L.A.R. Mutsaerts<br>Orthopaedic Surgeon and Unit Leader		15-05-2025
Project leader:<br>Dr. N.W. Willigenburg<br>Research coordinator		15-05-2025
Principal Investigator:<br>Prof. R.W. Poolman<br>Professor of orthopaedic surgery and Healthcare Evaluation		15-05-2025

TABLE OF CONTENTS

1.INTRODUCTION AND RATIONALE ... 8
2.OBJECTIVES ... 9
3.STUDY DESIGN ... 9
4.STUDY POPULATION ... 11
4.1Population (base) ... 11
4.2Inclusion criteria ... 11
4.3Exclusion criteria ... 11
4.4Sample size calculation ... 11
5.TREATMENT OF RESEARCH PARTICIPANTS ... 12
5.1Investigational product/treatment ... 12
5.2Use of co-intervention ... 12
5.3Escape medication ... 12
6.INVESTIGATIONAL PRODUCT ... 12
7.NON-INVESTIGATIONAL PRODUCT ... 12
8.METHODS ... 13
8.1Study parameters/endpoints ... 13
8.1.1Main study parameter/endpoint ... 13
8.1.2Secondary study parameters/endpoints ... 13
8.1.3Other study parameters ... 13
8.2Randomisation, blinding and treatment allocation ... 13
8.3Study procedures ... 13
8.4Withdrawal of individual research participants ... 16
8.5Replacement of individual research participants after withdrawal ... 16
8.6Follow-up of research participants withdrawn from treatment ... 16
8.7Premature termination of the study ... 16
9.SAFETY REPORTING ... 16
9.1Temporary halt for reasons of research participant safety ... 16
9.2AEs, SAEs ... 17
9.2.1Adverse events (AEs) ... 17
9.2.2Serious adverse events (SAEs) ... 17
10.STATISTICAL ANALYSIS ... 17
10.1Primary study parameter(s) ... 18
10.2Secondary study parameter(s) ... 18
10.3Cost-effectiveness ... 19
10.4Other study parameters ... 19
10.5Interim analysis ... 20

11.ETHICAL CONSIDERATIONS 20
11.1Regulation statement 20
11.2Recruitment and consent 20
11.3Objection by minors or incapacitated research participants (if applicable)..................21
11.4Benefits and risks assessment, group relatedness..21
11.5Compensation for injury 21
11.6Incentives..22
12.ADMINISTRATIVE ASPECTS, MONITORING AND PUBLICATION 22
12.1Handling and storage of data and documents 22
12.2Monitoring and Quality Assurance...22
12.3Amendments..23
12.4Annual progress report..23
12.5Temporary halt and (prematurely) end of study report..23
12.6Public disclosure and publication policy 23
13.STRUCTURED RISK ANALYSIS 23
14.REFERENCES..24

LIST OF ABBREVIATIONS AND RELEVANT DEFINITIONS

**AE**	**Adverse Event**
**CCMO**	**Central Committee on Research Involving Human Subjects; in Dutch: Centrale Commissie Mensgebonden Onderzoek**
**COD**	**Check-up On Demand**
**FU**	**Follow-Up**
**GCP**	**Good Clinical Practice**
**GDPR**	**General Data Protection Regulation; in Dutch: Algemene Verordening Gegevensbescherming (AVG)**
**HCP**	**Healthcare Provider**
**IB**	**Investigator’s Brochure**
**IC**	**Informed Consent**
**IMDD**	**Investigational Medical Device Dossier**
**METC**	**Medical research ethics committee (MREC); in Dutch: medisch-ethische toetsingscommissie (METC)**
**Review Committee**	**Medical research ethics committee (MREC) or CCMO**
**RFU**	**Routine Follow Up**
**(S)AE**	**(Serious) Adverse Event**
**SF**	**Short Form**
**Sponsor**	**The sponsor is the party that commissions the organisation or performance of the research, for example a pharmaceutical company, academic hospital, scientific organisation or investigator. A party that provides funding for a study but does not commission it is not regarded as the sponsor, but referred to as a subsidising party.**
**THA**	**Total Hip Arthroplasty**
**TKA**	**Total Knee Arthroplasty**
**UAVG**	**Dutch Act on Implementation of the General Data Protection Regulation; in Dutch: Uitvoeringswet AVG**
**WMO**	**Medical Research Involving Human Subjects Act; in Dutch: Wet Medisch-wetenschappelijk Onderzoek met Mensen**

SUMMARY

Rationale: Clinical guidelines recommend routine follow up (RFU) with X-ray and clinical visit at 1 year after Total Hip Arthroplasty (THA) and Total Knee Arthroplasty (TKA). However, evidence for (cost-)effectiveness of RFU is lacking. Registry data show excellent survival of THA and TKA and the added value of RFU can be questioned, as revisions are rare and seldom without symptoms. However, replacing RFU with a final follow up within 3 months and thereafter only check-ups on demand (COD), could lead to risks such as missed complications, lower patient satisfaction, or decreased physical function.

Objective: To investigate the (cost-)effectiveness of COD compared to RFU at 1 year, up to 2 years after THA and TKA.

Study design: A hybrid effectiveness (de-)implementation trial type II, with a stepped-wedge cluster trial design.

Study population: Patients scheduled for THA or TKA who experience pain and disability in the knee or hip joint due to osteoarthritis, and aged ≥ 50 years at the time of the surgery.

Intervention: Check-up on demand (COD) by the patient or healthcare provider (HCP), after a final clinical visit with X-ray within 3 months to diagnose complications and provide instructions when to contact an HCP.

Comparator: Routine follow up (RFU) consisting of an X-ray and clinical visit within 3 months and at 1 year after surgery. Patients are welcome to contact an HCP in case of pain, concerns or other symptoms associated with their prosthesis at any time.

Main study parameters/endpoints: The main study parameters are clinical and process outcomes. The clinical outcome is patient-reported physical function, measured with the PROMIS questionnaire, 2 years after surgery (1 year after RFU or COD). The process outcome is healthcare consumption, including X-rays and clinical visits.

Nature and extent of the burden and risks associated with participation: In the RFU group, the burden involves a hospital visit for an X-ray and clinical visit. In the COD group, the burden is lower, as participants attend follow-up visits only if necessary. However, there is a small risk of missed complications. This risk is considered minimal due to the low occurrence of asymptomatic complications. Studies and clinical practice show that when complications occur without pain, patients often do not take action, or this can lead to overdiagnosis and overtreatment. Participants will complete questionnaires pre-operative, 1 year after THA or TKA, 15, 18 and 24 months after THA or TKA. This burden is minimal, as only essential questions are included, developed in collaboration with patient representatives. Patient interviews showed no concerns about participation in this study.

1. INTRODUCTION AND RATIONALE

Total Hip Arthroplasty (THA) and Total Knee Arthroplasty (TKA) are two of the most common procedures for patients suffering from osteoarthritis and other degenerative joint diseases. These surgeries are highly effective in reducing pain and improving mobility. In 2022, more than 36,700 THAs and 26,700 TKAs were implanted (1,2), with osteoarthritis being the primary indication in 86% and 97% of cases, respectively (3,4). Given the growing elderly population and rising demand for these surgeries, the need for optimized follow up care has become increasingly important.

Current clinical guidelines recommend routine follow up (RFU) for patients after THA and TKA, with an X-ray and clinical visit within 3 months post-surgery and again at 1 year (5,6). There is currently insufficient high-level evidence to support or challenge the continued use of routine follow-up. The current guidelines for RFU after THA and TKA are based on limited observational data, and randomized controlled trials (RCTs) in this area are rare. A recent NIHR report recommended eliminating RFU before 1 and 10 years in patients with well-functioning implants but did not address the cost-effectiveness or other outcomes relevant to patients associated with RFU 1 year after surgery (7).

Studies show that early follow-up up (FU) to 1 year after surgery results in 0–4.6% detected abnormalities (8,9), of which only a small proportion impacts clinical decision-making and requires additional treatment (10-13). Moreover, in patients indicated for revision surgery, >96.7% present with clinical symptoms such as pain or loss of function. This suggests that early RFU could be safely replaced by check-ups on demand (COD). Registry data also indicate that revisions are rare and typically preceded by symptoms (14-16), bringing the added value of routine follow-up into doubt. Clinical experience further supports the notion that the risk of missed complications is low for asymptomatic patients, which raises the question of whether RFU can be safely de-implemented.

Despite this, eliminating RFU entirely is not without risks. The balance between reducing unnecessary healthcare resource use and ensuring patient safety requires careful consideration. Additionally, patients may find it reassuring to receive confirmation that everything is going well and that they can go back to normal activities, especially as many are not fully recovered at the clinical visit within 3 months post-surgery. Therefore, we suggest a final follow-up within 3 months and then offering COD, as requested by the patient or the healthcare provider (HCP).

The primary objective of this study is to assess whether RFU after THA and TKA can be safely replaced by COD, by comparing the cost-effectiveness of the two approaches 1 year after surgery. The HAKA trial will generate the high-quality evidence necessary to standardize care and guide future clinical practice.

2. OBJECTIVES

Primary Objective: At 2 years after surgery, what is the effect of a final follow up within 3 months combined with check-ups on demand (COD) compared to routine follow-up (RFU) within 3 months and at 1 year after total hip and knee arthroplasty (THA and TKA) on physical function (effectiveness) and clinical visits (implementation)?

Secondary Objective(s): At 2 years after surgery, what is the effect of a final follow up within 3 months combined with COD compared to RFU within 3 months and at 1 year after THA and TKA on complications, surgical interventions, quality of life (QoL), pain and costs.

3. STUDY DESIGN

This study has been developed in collaboration with stakeholders, as part of the Zorgevaluatie & Gepast Gebruik (ZE&GG) program. The stakeholders include clinicians, general practitioners, physiotherapists, patient representatives, epidemiologists, a cost-effectiveness expert, health insurers, guideline developers, and implementation experts. During a co-creation phase, multiple meetings were held in which the diverse interests and expertise of these stakeholders were integrated into the study design. Their continued involvement will guide the study's progress and implementation throughout the project.

This is a hybrid effectiveness (de-)implementation trial type II (17), with a stepped-wedge cluster trial design. A total of 10 hospitals will transition from a period with RFU to a period with COD, with a wash-out period (transition) of 2 months. The aim is to gradually de-implement RFU, with all centers eventually using COD as the standard of care. The inclusion period will last 14 months, followed by a 2-year follow-up for each patient. The total duration of data collection will be from July 2025 (first patient inclusion) to August 2028 (last patient, last visit).

Table 1. Monthly patient inclusion per center in a stepped wedge design.

Period	Center 1	Center 2	Center 3	Center 4	Center 5	Center 6	Center 7	Center 8	Center 9	Center 10	Total	
	Anticipated included patients											Cumulative
jul-25	4	4	4	4	4	4	4	4	4	4	40	40
aug-25	6	6	6	6	6	6	6	6	6	6	60	100
sep-25	10	10	10	10	10	10	10	10	10	10	100	200
oct-25	10	10	10	10	10	10	10	10	10	10	100	300
nov-25	Transition	Transition	10	10	10	10	10	10	10	10	80	380
dec-25	Transition	Transition	Transition	Transition	10	10	10	10	10	10	60	440
jan-26	10	10	Transition	Transition	Transition	Transition	10	10	10	10	60	500
feb-26	10	10	10	10	Transition	Transition	Transition	Transition	10	10	60	560
mar-26	10	10	10	10	10	10	Transition	Transition	Transition	Transition	60	620
apr-26	10	10	10	10	10	10	10	10	Transition	Transition	80	700
may-26	10	10	10	10	10	10	10	10	10	10	100	800
jun-26	10	10	10	10	10	10	10	10	10	10	100	900
jul-26	6	6	6	6	6	6	6	6	6	6	60	960
aug-26	4	4	4	4	4	4	4	4	4	4	40	1000
Total	100	100	100	100	100	100	100	100	100	100	1000	

Routine follow-up (RFU)
Check-Up On Demand (COD)

Note: The numbers per month represent the inclusion of patients with either THA or TKA, starting with 2 patients per month for each surgery, followed by 3 patients per month, and then 5 patients with THA and 5 patients with TKA per month.

The order in which the ten hospitals transition from RFU to COD will be determined through randomization. Randomization at the center level enhances the study's methodological rigor, reducing potential biases and increasing the generalizability and strength of the evidence. Besides, the study will be nested in the LROI database, which facilitates long term FU to monitor revision surgeries and mortality beyond the current projects timeline, with minimal additional effort.

4. STUDY POPULATION

4.1 Population (base)

The study population will consist of patients scheduled for primary Total Hip Arthroplasty (THA) or Total Knee Arthroplasty (TKA) due to osteoarthritis within the Netherlands. This population is drawn from a diverse source population, reflecting the broader demographic characteristics of patients undergoing these procedures in Dutch hospitals.

Patients undergoing THA and TKA are predominantly female, with more than 60% representation for both procedures. The average age of patients is approximately 70 years for THA and 68 years for TKA.

On average, each participating center performs 600 THA and 400 TKA procedures annually.

Given that, there is a high likelihood that the planned recruitment targets will be met. Additionally, the intervention is part of standard care regardless of whether the patient participates in the study, and the study burden for participants is minimal, further increasing the probability of successful recruitment.

4.2 Inclusion criteria

In order to be eligible to participate in this study, a participant must meet all of the following criteria:

- Painful and disabled hip or knee joint resulting from osteoarthritis
- Scheduled for primary THA or TKA surgery
- Age 50 years or older at the time of THA or TKA
- Capable and willing to complete questionnaires
- Proficient in Dutch or English
- Willing to provide informed consent

4.3 Exclusion criteria

A potential participant who meets any of the following criteria will be excluded from participation in this study:

- Other indication for surgery than osteoarthritis
- Scheduled for hip or knee revision arthroplasty, except a conversion from unicompartmental knee arthroplasty to TKA or from hip hemiarthroplasty/resurfacing to THA
- Already participating in this study due to a previous hip or knee surgery

4.4 Sample size calculation

We carefully considered the number of patients needed to obtain reliable estimates of the outcomes with the co-creating stakeholders and also asked orthopaedic surgeons for their opinion of convincing evidence. The agreed sample size is 250 patients per group. To provide a rough indication of the statistical power, we used the online power calculator tool available on Sealed Envelope (18). If there is truly no difference between COD and RFU, 190 patients (per intervention group) are required to be 90% sure that the lower limit of a one-sided 97.5% confidence interval will be above the non-inferiority limit of 3 on the PROMIS score with a standard deviation of 9. Anticipating 20-25% loss to follow up, we will include 250 patients per study intervention group (RFU and COD), thus a total of 500 THA and 500 TKA patients.

5. TREATMENT OF RESEARCH PARTICIPANTS

Routine Follow Up (RFU): X-ray and clinical visit following THA and TKA guidelines within

standard care: within 3 months and at 1 year after surgery. Patients are welcome to contact a HCP (i.e. general practitioner, physical therapist or hospital) in case of pain, concerns or other symptoms associated with their prosthesis at any time. RFU after THA and TKA is part of the guidelines “Totale heupprothese” and “Totale knieprothese” (5,6).
Check-Up on Demand (COD): final clinical visit with X-ray within 3 months and instructions how and when to contact a HCP. Further check-ups are scheduled only on demand (COD) by the patient or HCP. Patients are welcome to contact a HCP (i.e. general practitioner, physical therapist or hospital) in case of pain, concerns or other symptoms at any time (similar to current standard care).
In clinical practice, there is already variability in how follow-up after total hip or knee arthroplasty is conducted. Both RFU and variations of COD are utilized across different hospitals, with the choice of approach sometimes varying between individual orthopedic surgeons. The Dutch Orthopaedic Association (NOV) has confirmed that the interventions evaluated in this study are considered part of standard care.

5.1 Investigational product/treatment
Not applicable.

5.2 Use of co-intervention
Not applicable.

5.3 Escape medication
Not applicable.

6. INVESTIGATIONAL PRODUCT
Not applicable.

7. NON-INVESTIGATIONAL PRODUCT
Not applicable.

8. METHODS

8.1 Study parameters/endpoints

8.1.1 Main study parameter/endpoint

- Clinical: PROMIS physical function
- Process (healthcare consumption): number of clinical visits and X-rays

8.1.2 Secondary study parameters/endpoints

- Number and type of complications (e.g. infection, peri-prosthetic fracture, loosening, malalignment or malposition of components, prosthetic wear, dislocation, etc.)
- Number and type of surgical interventions (e.g. DAIR, partial component exchange, revision surgery (full prosthesis replacement), irrigation and debridement (without component retention), etc.)
- Additional healthcare consumption related to THA or TKA FU (e.g. clinical visit, telephone consultations, X-ray, CT scan, MRI scan, laboratory tests, other)
- Health related quality of life (EQ-5D-5L)
- Numeric Pain Rating Scale (NPRS)
- Numeric Satisfaction Rating Scale (NSRS)
- Costs (based on electronic patient records and additional questionnaires, including all visits to healthcare providers outside the hospital)

8.1.3 Other study parameters

- Patient characteristics (e.g. age, gender, BMI, ASA score, ethnicity, employment status, and education level).
- Surgical characteristics (e.g. surgical approach, prosthesis type and fixation method, surgical duration, and intra-operative complications)
- Postoperative characteristics (e.g. length of hospital stay, in-hospital complications and discharge destination)
- Healthcare consumption, complications, and surgical interventions from the time of THA/TKA up to one year postoperatively

8.2 Randomisation, blinding and treatment allocation

Treatment allocation will occur naturally through the stepped-wedge design of the study. All participating centers will initially use routine follow-up (RFU) and will transition to check-up on demand (COD) at predetermined points during the study

8.3 Study procedures

Patients scheduled for total THA or TKA will be invited to participate in the study prior to their surgery. This timing ensures that participation is not influenced by the surgery itself, and a patient's potential preference for follow-up care.
Information on patient background will be collected to explore potential differences in follow-up needs, including among patients with different ethnic backgrounds. Previous research shows that people with a migrant background visit specialists and hospitals less often than

native Dutch patients with similar health and SES (19). Acknowledging the sensitivity of this topic, a ‘prefer not to say’ option is provided.

If the center where the patient undergoes surgery is in RFU phase, follow-up visits will be scheduled within 3 months and 1 year postoperatively. If the center is in the COD phase, patients will receive instructions during their 3-month follow-up on when and how to contact a HCP in case of concerns or complaints. In the COD phase, no standard 1-year follow-up visit will be scheduled.

Participants will be followed up to 2 years after surgery, i.e. 1 year after RFU or COD. The primary outcome, costs and the EQ-5D-5L will be measured pre-operative, at 1 year after surgery and at 15, 18 and 24 months after surgery. The other outcomes will be measured pre-operative (baseline) and at 1 year and 2 years after surgery (endpoint). Figure 1 depicts a flowchart of study participation.

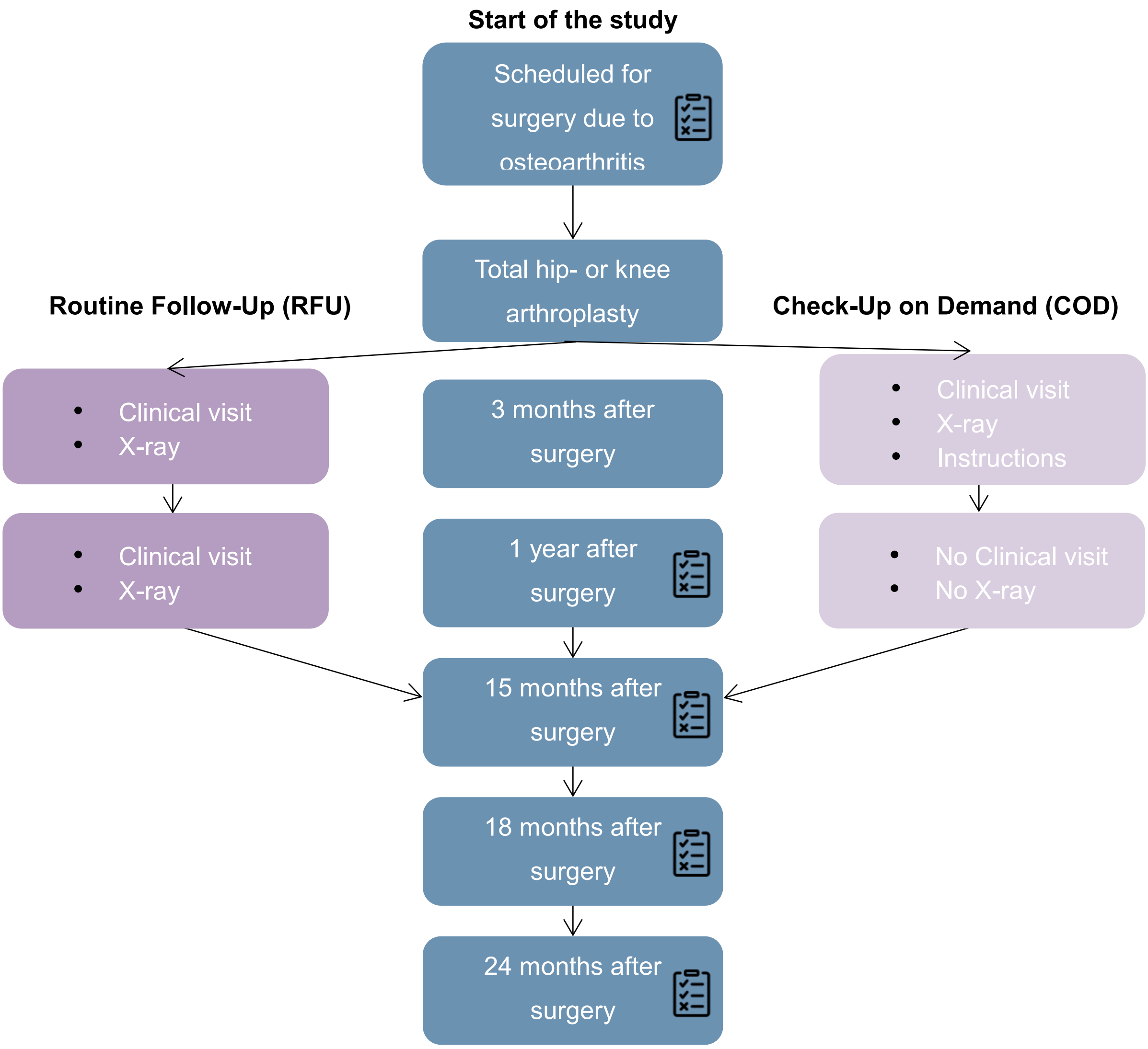

Figure 1. Flowchart of study participation.

All participants will be asked to complete the questionnaires online or on paper, this will take around 30 minutes. Table 2 shows which assessments will be made at which times during follow-up. Study data will be collected in the patient's electronic file and will finally be collected onto an electronic case report form (eCRF) in Castor EDC.

Table 2. Overview of the study procedures and assessments at each time point.

	Before surgery	Surgery	Discharge	3 months after surgery	1 year* after surgery: RFU or COD	15 months* after surgery	18 months* after surgery	24 months* after surgery
ICF procedure	X							
Patient characteristics	X							
Surgical characteristics		X						
Postoperative characteristics			X					
Clinical visit				X	X (for RFU)			
X-ray				X	X (for RFU)			
Complications								X
Surgical interventions								X
Healthcare consumption								X
PROMIS	X				X	X	X	X
EQ-5D-5L	X				X	X	X	X
NPRS	X				X			X
NSRS					X			X
Costs						X	X	X

***A maximum window of ±30 days will be applied for data collection of the questionnaires.**

PROMIS physical function will be obtained using computer-adaptive testing (CAT). If this is not feasible, the short form (SF) can be completed either digitally or on paper. Version 10a will be used. The SF PROMIS physical function consists of 10 questions and is expressed by raw summed score ranging from 10 to 50, which can be converted to a T-score and SE. The T-score is a standardized score with a mean of 50 and a SD of 10.

Health related quality of life (EQ-5D-5L) is a general health-related quality of life questionnaire and consists of five questions regarding mobility, self-care, usual activities, pain/discomfort and anxiety/depression and one visual analog scale (VAS) to document the perceived quality of life. The quality of life is described by 2 scores, the index value, which range from 0 to 1, and the VAS score, which range from 0 to 100. For both scores applies the higher the better.

Numeric Rating Scale for pain (NPRS) is a scale ranging from zero to ten on which patients can score their pain. It is a widely used instrument in varying populations due to its ease of administration and clinical relevance to the patient. The lower the score, the better.

Numeric Rating Scale for satisfaction (NSRS) will be measured by a single question: “How satisfied are you with the results of the treatment?” The patient will answer this question with a NRS scale ranging from zero to ten. The higher the score, the better.

Health consumption and costs will be measured using the EPD. Health consumption includes both the primary outcomes clinical visits and X-rays, as well as additional health care use, including telephone consultations and scans. The LROI register will be used to collect data about revision surgery. A questionnaire will be used to get more insight in the health consumption and costs which cannot be obtained from the EPD. This questionnaire is made in collaboration with a HTA expert and patient representatives.

8.4 Withdrawal of individual research participants

Participants can leave the study at any time for any reason without any consequences if they wish to do so. The investigator can decide to withdraw a participant from the study for urgent medical reasons.

8.5 Replacement of individual research participants after withdrawal

Replacement of individual subjects after withdrawal is not applicable, as the power calculation has already accounted for potential loss to follow-up.

8.6 Follow-up of research participants withdrawn from treatment

If a participant chooses to withdraw from the study, all data collected up to the point of withdrawal may still be used for analysis, unless the participant explicitly requests otherwise.

8.7 Premature termination of the study

As both COD and RFU are part of standard care, premature study termination is not expected unless significant safety concerns arise. If the study is terminated early, subjects will continue follow-up according to the standard care procedures at their hospital. Subjects will not face any additional risks if the study is prematurely terminated. All relevant parties will be notified, and data collected up until termination will be securely managed and analyzed. A final report outlining the reasons for termination and the findings will be provided to the relevant stakeholders.

9. SAFETY REPORTING

9.1 Temporary halt for reasons of research participant safety

In accordance to section 10, subsection 4, of the WMO, the sponsor will suspend the study if there is sufficient ground that continuation of the study will jeopardise participant health or safety. The sponsor will notify the review committee without undue delay of a temporary halt including the reason for such an action. The study will be suspended pending a further positive decision by the review committee. The investigator will take care that all participants are kept informed.

9.2 AEs, SAEs

9.2.1 Adverse events (AEs)

Adverse events are defined as any undesirable experience occurring to a participant during the study. In this study, complications, surgical interventions, and healthcare use are systematically collected as part of the study outcomes and are therefore already recorded. As such, these do not need to be reported separately as adverse events. Only events that can be directly linked to the presence or absence of the clinical visit and X-ray 1 year after surgery should be reported as adverse events.

9.2.2 Serious adverse events (SAEs)

A serious adverse event is any untoward medical occurrence or effect that

- results in death;
- is life threatening (at the time of the event);
- requires hospitalisation or prolongation of existing inpatients' hospitalisation;
- results in persistent or significant disability or incapacity;
- is a congenital anomaly or birth defect; or
- any other important medical event that did not result in any of the outcomes listed above due to medical or surgical intervention but could have been based upon appropriate judgement by the investigator.

Only events that are related to the study procedures (RFU or COD) will be considered as serious adverse events. An elective hospital admission will not be considered as a serious adverse event.

Due to the low risk of the interventions (COD or RFU), which are both already part of standard care, hardly any SAEs are to be expected. This means all (S)AEs related to

participation in this study protocol, meaning from COD and RFU, will not be reported to the METC, as no patient benefit is expected from this.

10. STATISTICAL ANALYSIS

All analyses will be performed separated for THA and TKA and will be performed using SPSS or R. Categorical data will be presented as numbers and percentages. Continuous variables will be presented by mean and standard deviation in case of a normal distribution. In case of non-normality, median and interquartile range will be given.
Missing data will be minimized as much as possible by contacting patients in case of incomplete questionnaires. After finalizing the database, the extent of missing data will be assessed. Mixed models will address missing data, and if necessary, multiple imputation will be employed, provided that the data are missing at random (20,21).

10.1 Primary study parameter(s)

PROMIS physical function will be presented as mean with standard deviation, assuming normality. Differences in PROMIS physical function between the RFU and COD groups will be compared using linear mixed models, with repeated measurements clustered within participants and participants clustered within hospitals (random intercepts). The intervention group (RFU vs. COD), baseline PROMIS physical function, and time points (12, 15, 18 and 24) will be included as fixed effects to determine the crude effect of the intervention at each time point, with the primary outcome at 24 months. Given the stepped wedge design, the duration of the intervention period differs between sites. Therefore “period” is also included as a fixed effect. To assess of the effect of COD might increase over a longer period of time, an interaction term (period * intervention) will be tested for these possible time effects for the primary outcome.
For healthcare consumption, the percentage of patients who have had at least one clinical visit or X-ray 1 year after RFU or COD will be presented for each group. Additionally, the average number of visits and X-rays per patient will be reported.
A logistic generalized linear mixed model (GLMM) will be used to compare the presence or absence of visits/X-rays (yes/no per patient) between RFU and COD. A Poisson regression analysis will be performed to assess the average number of visits and X-rays per patient, adjusting for potential confounders. Interaction terms between the confounders and the intervention group will also be included to explore whether certain patient characteristics influence the differences in healthcare consumption between RFU and COD.

10.2 Secondary study parameter(s)

For complications, surgical interventions, and additional healthcare consumption, the percentage of patients who have experienced each will be presented for each group.

Additionally, the total number of complications, interventions, and additional healthcare consumption per patient will be reported. Logistic GLMM will be used to compare the presence or absence of complications, surgical interventions, and additional healthcare consumption (yes/no per patient) between RFU and COD. A Poisson regression analysis will be performed to assess the total number of complications, interventions, and additional healthcare consumption per patient, adjusting for potential confounders.
Quality of life (EQ-5D-5L) and pain (NPRS) will be analysed using mixed models, with repeated measures clustered within participants and participants within hospitals (random intercepts). Intervention group (RFU vs. active COD vs. passive COD), baseline score, and time (15, 18 and 24 months) are included as fixed effects. Satisfaction (NSRS) will be analyzed using independent t test to compare the mean difference between the RFU and COD group.

10.3 Cost-effectiveness

The trial-based economic evaluations will focus on the primary outcome (PROMIS physical function) and QALYs, following the intention-to-treat principle. Two perspectives will be considered: 1) healthcare and 2) societal. QALYs will be calculated by multiplying utility values (based on EQ-5D-5L and valued using the Dutch tariff) by health state durations (22,23). Resource utilization will be assessed via cost questionnaires (15, 18 and 24 months after surgery) and valued according to the “Dutch manual of costing” (24). Missing data will be handled with multivariate imputation by chained equations (25). Societal costs include healthcare, absenteeism, presenteeism, unpaid productivity, and informal care; healthcare costs cover only the formal Dutch healthcare sector. Linear mixed models will estimate cost and effect differences, accounting for clustering (e.g., hospital level). Incremental cost-effectiveness ratios (ICERs) will be calculated as cost differences divided by effect differences, with uncertainty assessed through non-parametric bootstrapping nested within multiple imputation. Results will be visualized in cost-effectiveness planes and acceptability curves (26,27). Sensitivity analyses, including complete-case analysis, will test result robustness (23). The budget impact analysis (BIA) will use ZonMw’s BIA tool, based on Dutch incidence data. Perspectives include societal, government (Budget Kader Zorg), and insurer. Scenarios include: 1) RFU-only implementation, 2) COD for all patients, and 3) COD for specific subgroups (e.g., low-complication risk), defined by study results. Costs will be valued using Dutch standard costs, tariffs set by the Dutch Healthcare Authority (NZA), or average insurer tariffs. The cost-effectiveness and BIA analyses will adhere to the 'Dutch guideline for economic evaluations in health care' (28).

10.4 Other study parameters

The additional study parameters, including patient characteristics, surgical characteristics, and postoperative characteristics, will be used to describe the study population and adjust for potential confounders. These variables will help account for potential baseline differences between groups, allowing for more accurate estimation of intervention effects. Patient characteristics, surgical characteristics, and postoperative characteristics will be presented as means and standard deviations for continuous variables, and frequencies and percentages for dichotomous variables. These characteristics will be used to describe the study population and adjust for potential confounders in the analysis. Including these variables in the model will help control for baseline differences between groups, thereby reducing bias and leading to more accurate and reliable estimates of the intervention effects.

10.5 Exploratory analysis

As an exploratory analysis, we will assess subgroup effects on the primary outcomes of complications, clinical visits and X-rays. The GLMMs described above will be extended, by including interaction terms for age, gender, BMI, ASA-score, ethnicity, employment status, and educational level with the intervention in separate models.

10.6 Interim analysis

Not applicable.

11. ETHICAL CONSIDERATIONS

11.1 Regulation statement

The study will be conducted in accordance with the principles of the Declaration of Helsinki (version 8, October 2024). In addition to this declaration, the research will comply with the Wet medisch-wetenschappelijk onderzoek met mensen (WMO), ensuring a legal framework for medical research involving human subjects. Adherence to Good Clinical Practice (GCP) guidelines will uphold high ethical standards. The Algemene Verordening Gegevensbescherming (AVG) will protect the privacy and personal data of participants. Finally, the Nederlandsche Code voor Wetenschappelijke Integriteit will guide ethical research practices. These frameworks will collectively ensure the rights, safety, and well-being of participants throughout the study.

11.2 Recruitment and consent

Hard-copy procedure: The recruitment process in each participating hospital will involve monthly outreach to patients scheduled for THA or TKA. The number of patients approached (2, 3, or 5) will depend on the hospital's position in the stepped-wedge schedule and the specific month of recruitment. To minimize selection bias, each consecutive patient, based on the scheduled date and time of surgery, will be asked to participate until the target

number of inclusions for that month is reached. The initial contact regarding the study will be made by the treating physician, by asking permission if he/she can be approached for the study. A researcher will contact the patient by phone, or at the outpatient department to assess their suitability for participation based on their medical history and the study's inclusion and exclusion criteria. They will then provide information about the study and ask whether the patient is interested in participating. The patient will receive the written patient information letter and informed consent form (Appendix X) and will be contacted by the research team after at least 5 days. The researcher will discuss the written patient information and answer any questions. Once all questions have been addressed, the patient will sign the informed consent form twice and return it. After receiving the signed form, the researcher will also sign it twice and send the patient one fully signed copy.

E-consent procedure: In this study, the recruitment process will also offer an e-consent option using Castor EDC, which securely collects and stores data in accordance with Good Clinical Practice (GCP). Access to the Castor EDC study page will be restricted to the study investigators and will include an audit trail to track data entries with timestamps and editor details. The initial stage of recruitment is the same as the hard-copy procedure; however, if the patient is interested in participating, the physician or researcher will send an email containing a personalized URL. By clicking the URL, patients will be directed to an introductory screen in Castor EDC, where the patient information letter is provided. Here, it is explained how a participant can revoke their consent during the study. Similar to the hard-copy procedure, the patient will be contacted by the research team after at least 5 days. The researcher will discuss the digital patient information letter and answer any questions. Once all questions have been addressed, the patient can provide consent using a Castor EDC survey presented at the next page after the digital patient information letter. Through locked checkboxes (yes/no), the patient can grant permission to store their data for use in other research, agree to be contacted one year after surgery for a focus group discussion, and consent to be asked about participation in follow-up studies (similar to the hard-copy procedure). Additionally, the patient will select the date they digitally signed the consent. The consent procedure is complete only if all boxes are selected. The participant will receive a copy of this informed consent form from the researcher by email.

11.3 Objection by minors or incapacitated research participants (if applicable)

Not applicable.

11.4 Benefits and risks assessment, group relatedness

Current clinical guidelines regarding RFU are based on low-level evidence, primarily from observational studies, raising questions about the necessity and value of these follow-up visits. By conducting this trial, we aim to fill the critical knowledge gap regarding the efficacy

and potential risks associated with de-implementing RFU in favor of a more patient-centered approach, such as COD. The benefits of participating in this study include a potentially reduced burden on patients, as they would only need to attend follow-up visits when necessary, thereby increasing convenience and satisfaction. Moreover, the study will generate high-level evidence to inform clinical guidelines, which can lead to more effective and tailored care for patients undergoing THA and TKA. However, there are risks associated with this shift in follow-up practice, such as the possibility of missed complications, particularly asymptomatic issues that may not prompt patient-initiated contact with healthcare providers. Despite this concern, the risk of serious complications occurring without accompanying symptoms is low, and the current literature indicates that many patients do not seek care when complications arise. The study's design, incorporating patient-reported outcomes and healthcare utilization data, will facilitate a thorough evaluation of the balance between the benefits of reduced follow-up burden and the risks of potential complications. Ultimately, this research will provide valuable insights into optimizing postoperative care, ensuring patient safety while promoting efficient healthcare practices.

11.5 Compensation for injury

The sponsor/investigator has a liability insurance which is in accordance with article 7 of the WMO. The METC gives dispensation for research participant insurance, because no additional risks are expected as RFU and COD are both part of standard care.

11.6 Incentives

In this study, no special incentives, compensation, or treatment will be provided to participants for their involvement. Participation is voluntary, and patients will not receive any financial or material benefits for taking part in the study.

12. ADMINISTRATIVE ASPECTS, MONITORING AND PUBLICATION

12.1 Handling and storage of data and documents

Data will be handled confidentially in accordance with Algemene Verordening Gegevensbescherming (AVG) and the Uitvoeringswet AVG (UAVG). We will collect data using the Castor Electronic Data Capture system, which ensures secure storage and management of personal data. Access to the source data will be limited to authorized personnel only, including the investigators and relevant research team members. Data will be coded to protect participants' identities, utilizing a subject identification code list that does not include patient initials or birth dates. The key to the code will be safeguarded at the local center and will not leave this location. All questionnaire items may be entered directly into Castor on an incidental basis, for example, if participants strongly prefer completing

questionnaires via telephone or if missing responses need to be clarified through follow-up calls.

Upon project completion, data will be stored in an appropriate repository or archive, ensuring that it is findable and accessible for future research. Data will be retained for 15 years after the completion of the study, in accordance with the guidelines of the Centrale Commissie Mensgebonden Onderzoek (CCMO). We will publish metadata, including the study protocol, data management plan, statistical analysis plan, data dictionary, and syntaxes, to facilitate reuse for other research purposes. An identifier (DOI) will be generated, and reuse conditions will be explicitly defined in the data management plan. In line with the principles of Open Science, we are committed to enhancing the findability, accessibility, interoperability, and reusability (FAIR) of our data. We will publish our findings in open-access journals to promote transparency, accessibility, and collaboration within the scientific community, thereby advancing orthopedic care and contributing to discussions on arthroplasty follow-up protocols.

12.2 Monitoring and Quality Assurance

The monitoring plan includes provisions for one initiation visit, one interim monitoring visit, and one close-out monitoring visit, along with travel expenses. Remote or digital monitoring may also be conducted as needed. The frequency of interim monitoring visits depends on the inclusion rate, though annual monitoring is typical.

12.3 Amendments

Amendments are changes made to the research after a favourable opinion by the review committee has been given. All amendments will be notified to the review committee that gave a favourable opinion. Non-substantial amendments will not be notified to the review committee, but will be recorded and filed by the sponsor.

12.4 Annual progress report

The sponsor/investigator will submit a summary of the progress of the trial to the review committee once a year. Information will be provided on the date of inclusion of the first participant, numbers of participants included and numbers of participants that have completed the trial, serious adverse events, other problems, and amendments.

12.5 Temporary halt and (prematurely) end of study report

The investigator/sponsor will notify the review committee of the end of the study within a period of 8 weeks. The end of the study is defined as the last patient’s last visit. The sponsor will notify the review committee immediately of a temporary halt of the study, including the reason of such an action. In case the study is ended prematurely, the sponsor will notify the review committee within 15 days, including the reasons for the premature termination.

Within one year after the end of the study, the investigator/sponsor will submit a final study report with the results of the study, including any publications/abstracts of the study, to the review committee.

12.6 Public disclosure and publication policy

In accordance with the requirements of the International Committee of Medical Journal Editors, the study will be registered in a public trial registry prior to the recruitment of the first patient. The results will be published in peer-reviewed scientific journals and presented at relevant (inter)national congresses. The arrangements for public disclosure and publication of the research data for the overall HAKA project are outlined in the ZonMw consortium agreement between the sponsors of the individual studies. For the participating centers within the studies, publication policies are specified in the Participation Agreements.

13. STRUCTURED RISK ANALYSIS

Not applicable.

14. REFERENCES

1. Dutch Arthroplasty Register (LROI). Hip arthroplasty: Numbers. Online annual report. 2022;13.
2. Dutch Arthroplasty Register (LROI). Knee arthroplasty: Numbers. Online annual report. 2022;77.
3. Dutch Arthroplasty Register (LROI). Total hip arthroplasty: Demographics. Online annual report. 2022;16.
4. Dutch Arthroplasty Register (LROI). Knee arthroplasty: Patient characteristics. Online annual report. 2022;81.
5. Federatie Medisch Specialisten. Totale heupprothese (THP). 2019.
6. Federatie Medisch Specialisten. Totale knieprothese (TKP). 2021.
7. Kingsbury SR et al. Safety of disinvestment in mid-to late-term follow-up post primary hip and knee replacement: the UK SAFE evidence synthesis and recommendations. Health and Social Care Delivery Research. 2022.
8. Hart AA et al. Routine radiographs after total joint arthroplasty. The Journal of Arthroplasty. 2021.
9. Christensen M, Folkmar K. No clinical value of post-operative routine X-ray following uncomplicated cementless primary total hip arthroplasty. Dan Med J. 2013.
10. Lovelock TM, Broughton N. Follow-up after arthroplasty of the hip and knee. The Bone & Joint Journal. 2018.
11. Paul HY et al. Do serologic and synovial tests help diagnose infection in revision hip arthroplasty? Clinical Orthopaedics and Related Research. 2015.
12. Hendricks TJ et al. The cost of routine follow-up in total joint arthroplasty and the influence of these visits on treatment plans. Kansas journal of medicine. 2018. > 12
13. Bessette MC et al. The utility of postoperative radiographs 2 years after primary total knee arthroplasty. The Journal of Arthroplasty. 2017.
14. Evans JT et al. How long does a knee replacement last? The Lancet. 2019.
15. Hacking C et al. Is there a need for routine follow up after primary total hip arthroplasty? ANZ journal of surgery. 2010
16. Cassidy R et al. Guidelines for the follow-up of total hip arthroplasty. The bone & joint journal. 2019.
17. Wolfenden et al. Designing and undertaking randomised implementation trials: guide for researchers. Bmj. 2021.
18. Sealed Envelope Ltd. Power calculator for continuous outcome non-inferiority trial [Internet]. London: Sealed Envelope Ltd.; 2001 [cited 2025 Apr 10].
19. van Berkum MTM, Smulders EMC. Migranten, preventie en gezondheidszorg [Internet]. Utrecht: Pharos; 2010 [cited 2025 Apr 8].
20. Austin PC et al. Missing Data in Clinical Research: A Tutorial on Multiple Imputation. Can J Cardiology. 2021.
21. Bhaskaran K, Smeeth L. What is the difference between missing completely at random and missing at random? Int J Epidemiology. 2014.
22. Versteegh MM et al. Dutch tariff for the five-level version of EQ-5D. Value in health. 2016.
23. Drummond MF et al. Methods for the economic evaluation of health care programmes. Oxford university press; 2015.
24. Kanters TA et al. Update of the Dutch manual for costing studies in health care. PloS one. 2017.

25. White IR et al. Multiple imputation using chained equations: issues and guidance for practice. Statistics in medicine. 2011.
26. Black WC. The CE plane: a graphic representation of cost-effectiveness. Medical decision making. 1990.
27. Fenwick E et al. Cost-effectiveness acceptability curves–facts, fallacies and frequently asked questions. Health economics. 2004.
28. Versteegh M et al. From good to better: new Dutch guidelines for economic evaluations in healthcare. Pharmacoeconomics. 2016.
