# Supplementary material for: Is check-up on demand non-inferior to routine follow-up at one year after total hip or knee arthroplasty in terms of clinical outcomes and cost-effectiveness? Protocol for a randomized stepped-wedge hybrid effectiveness de-implementation trial
Source: PLoS One. 2026 Mar 17;21(3):e0343627. doi: 10.1371/journal.pone.0343627 (PMC12994803; doi:10.1371/journal.pone.0343627)
Supplement: S5 File — (PDF) [file pone.0343627.s005.pdf]

Roles of patient engagement per study phase.

		ROLE IN PROJECT				
Phase	Activities	Listener<br>*Is given information*	Co-thinker<br>*Is asked to give opinion*	Advisor<br>*Gives (un) solicited advice*	Partner<br>*Works as an equal partner*	Decision-maker<br>*Takes initiative, (final) decision*
Preparation	Project meetings	Attending project meetings		Assessing feasibility, patient burden, and safety of the study; refining the research question<br><br>Safeguarding patient-relevant outcomes	Determining patient recruitment strategy<br><br>Deciding on the primary outcome	
	Patient information	Reviewing different versions of the patient information letter and consent form		Advising on the relevance, readability, and clarity of the information letter and consent form<br><br>Contributing to instruction video for patient participation		
	Design of the follow-up within 3 months			Advising on the information patients receive<br><br>Contributing to the instruction video for 3-month follow-up	Co-deciding on the setup of the 3-month check-up. When should patients contact their (general) practitioner?	
	Questionnaires		Testing questionnaires (feasibility, time investment)		Discussing the selection of patient-reported outcome measures	

Execution	Conducting the study	Receiving monthly newsletters	Practicing inclusion conversation: amount of information and decision-making burden	Advising on patient recruitment strategies if inclusion is below expectations	Co-determining solutions to general bottlenecks<br><br>Co-deciding during project group meetings	
	Focus groups		Participating in a focus group with patient partners		Recruiting and supporting focus group participants<br><br>Formulating questions for patient discussions	
	Patient newsletter				Co-developing the newsletter with the research team	Sharing updates with their networks
	Data analysis		Contributing to the interpretation of study results	Advising on the feasibility of conclusions and patient-relevant recommendations		
	Reporting			Advising on which study findings are relevant to patients	Co-deciding on the content of the lay summary	
Implementation	Dissemination of results				Making content accessible for patients prior to dissemination	Sharing interim and final results via the websites and social media
	Publications / presentations	Receiving study summaries and attending project debriefings			Writing an article for the patient organization magazine<br><br>Reviewing scientific output	Presenting results to patient communities via webinar, podcast, or conference
	Evaluation			Evaluating the project process and sharing strengths and areas for improvement		

	Guideline updates			Contributing input for updates to clinical guidelines by participating in the guideline working group		

Source: www.participatiematrix.nl
