# Supplementary material for: Is check-up on demand non-inferior to routine follow-up at one year after total hip or knee arthroplasty in terms of clinical outcomes and cost-effectiveness? Protocol for a randomized stepped-wedge hybrid effectiveness de-implementation trial
Source: PLoS One. 2026 Mar 17;21(3):e0343627. doi: 10.1371/journal.pone.0343627 (PMC12994803; doi:10.1371/journal.pone.0343627)
Supplement: S6 File — (PDF) [file pone.0343627.s006.pdf]

What to do with hip replacement complaints?

You have received a hip prosthesis ("hip replacement"). Usually, recovery goes well, but it can take up to a year for your body to fully adjust to the prosthesis. Problems with the prosthesis occur in about 1-3% of people. This flyer helps you recognize what is normal and explains when and how to contact us if you experience any issues.

What is normal?

- Pain in your thigh, buttock, or groin that gets better gradually
- Stiffness around the hip after standing up or prolonged periods of rest

What is not normal?

New complaints that **suddenly appear** and last longer than 2 weeks or worsen, such as:

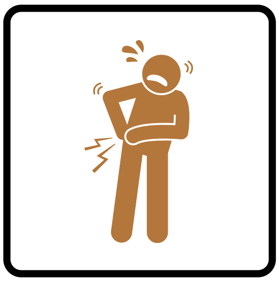
Pain

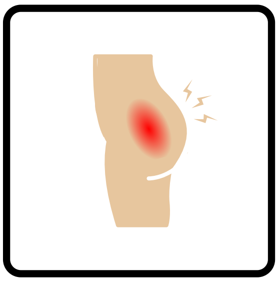
Warmth or redness

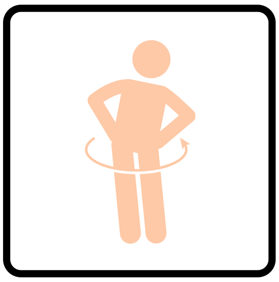
Feeling of instability or buckling

What to do?

Do you have one of these “abnormal” complaints or are you unsure?

Call the Orthopedics outpatient clinic at [name hospital] at [phone number] or email us at [email address]

[*Logo of the participating hospital*]

What to do with knee replacement complaints?

You have received a knee prosthesis ("knee replacement"). Usually, recovery goes well, but it can take up to a year for your body to fully adjust to the prosthesis. Problems with the prosthesis occur in about 1-3% of people. This flyer helps you recognize what is normal and explains when and how to contact us if you experience any issues.

What is normal?

- Stiffness or a band-like feeling around the knee that slowly gets better
- Clicking or cracking sounds from the knee
- Difficulty kneeling or squatting

What is not normal?

New complaints that **suddenly appear** and last longer than 2 weeks or worsen, such as:

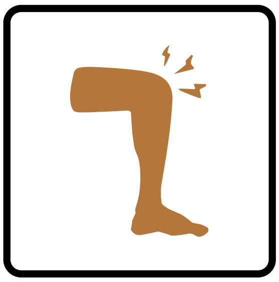

Pain

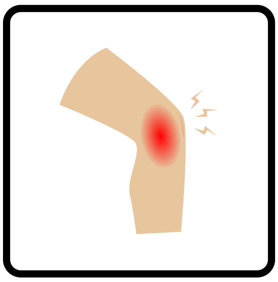

Warmth or redness

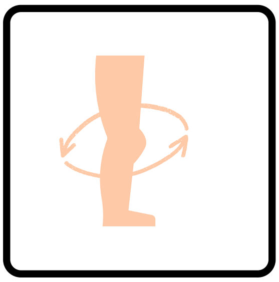

Feeling of instability or buckling

What to do?

Do you have one of these “abnormal” complaints or are you unsure?

Call the Orthopedics outpatient clinic at [*name hospital*] at [*phone number*] or email us at [*email address*]

HAKA

[*Logo of the participating hospital*]
