# Supplementary material for: Is check-up on demand non-inferior to routine follow-up at one year after total hip or knee arthroplasty in terms of clinical outcomes and cost-effectiveness? Protocol for a randomized stepped-wedge hybrid effectiveness de-implementation trial
Source: PLoS One. 2026 Mar 17;21(3):e0343627. doi: 10.1371/journal.pone.0343627 (PMC12994803; doi:10.1371/journal.pone.0343627)
Supplement: S7 File — (PDF) [file pone.0343627.s007.pdf]

ZONMW (NEDERLANDS): DATA MANAGEMENT HAKA (ZONMW-TEMPLATE 2019)

1. GENERAL FEATURES OF THE PROJECT AND DATA COLLECTION

1.1 Project leader contact details

Prof. R.W. Poolman
Professor in Healthcare Evaluation and Orthopaedic surgeon
Department of Orthopaedics, OLVG

1.2 I have composed my DMP with the assistance of a data stewardship (or management) expert. List his or her name, function, organization/department, phone number and email address.

MSc. I.K. Butter
Science Advisor in Data Management
Teaching Center, OLVG

- The data expert has been trained (and has certification) in data stewardship (please explain) → The data expert is well-trained in data stewardship and has solid experience in managing and protecting data. She knows how to ensure data accuracy, privacy, and compliance with regulations.
- The expert is connected to my department or institution (please explain his/her expertise related to data stewardship) → The data expert is a key member of our "Science Team" at OLVG, specializing in data stewardship. They ensure effective data management, maintain data quality, and support researchers in best practices, thus enhancing our institution's research and data governance.
- The expert is not connected to my department or institution (please explain his/her expertise related to data stewardship)

1.3 In collecting data for my project, I will do the following:

- Add new data to an existing data set (please specify)
- Merge different data files (please specify)
- Use an MDS (Minimal Data Set)
- Use existing data (please specify) → We will use existing data from the National Joint Registry (LROI) to enable long term FU (i.e. revision or death) of participants (beyond the timeline of our project) and to select eligible patients for WP2, but only when it is not possible to identify these patients using alternative methods within the hospital.
- Generate new data

Guidance:
You may find (references to) existing databases in publications, catalogues, archives or data platforms. The guidance theme 'Data sharing' has a list of some well-known resources.

1.4 In my research, I will use:

- A combination of quantitative and qualitative data
- Exclusively qualitative data
- Exclusively quantitative data
- Other (please specify)

Guidance:
For both quantitative and qualitative research it is important to create FAIR data. That enables other researchers to verify the outcomes of your research, and / or reuse the data for new research.
Research data management and stewardship differs in some aspects for quantitative and qualitative research.

It is ZonMw's intention to accommodate both types of research in this DMP template. In case you come across conflicting or missing items, please explain in the DMP or contact openscience@zonmw.nl

1.5 I will be reusing or combining existing data, and I have the owner's permission for that.

- Yes, I have permission to use the data, but I am required to destroy them at the end of the project
- Yes, I have permission to use the data
- No permission is required, since the data are openly accessible → Hospitals can use data related to patients under their own care, so the hospital is considered the 'owner' of the data, provided that the data are used exclusively within the institution and are not shared externally.
- No, I will not be reusing or combining existing data

Guidance:
When using existing data consider:

- whether conditions apply to the use of the data and whether conflicts could arise with existing research;
- the fact that for the reproducibility of results you need to note which version of the existing data sets you use.
- the available formats of the dataset and to choose the one that fits best (consider e.g., limitations, restrictions for use)
- convert or translate different standards in pre-existing data sets in order to link or enhance them.

More information in DS wizard (Is there pre-existing data?)

1.6 In collecting new data, I will be collaborating with other parties.

- Yes, we have reached agreements on the user rights of the data in the project through a 'Samenwerkingsovereenkomst' according to the Consortium Agreement Template for ZonMw funded Projects.
- Yes, I will collect the new data in conjunction with other researchers or research groups.
- No
- Yes, the new data will be (partly) provided by a project partner or supplier

Guidance:
If you are collaborating with other organisations on the gathering of research data, it is important to:

- make clear arrangements concerning the accessibility, reusability, exchangeability and verifiability of the new data set;
- **agree** on **ownership or co-producership** of data;
- draw up **terms and conditions** for the use of data by third parties (e.g. co-authorship, permission for research, information on new research reports and papers);
- allocate **responsibilities** in the research process;

- record all these arrangements in writing in the form of a **collaboration or consortium agreement.**

→ We will address these aspects by making Clinical Trial Agreements (CTAs) and a Consortium agreement.

For more information on ZonMw public-private partnership and co-funding: Grants and Collaborations/contributions from third parties | ZonMw

1.7 I am a member of a consortium of 2 or more partners. Clear arrangements have been made regarding data management and intellectual property (also consider the possible effect of changes within the consortium on issues of data management and intellectual property).

- Yes, I am a member of a consortium of 2 or more partners, but clear arrangements have not (yet) been made regarding data management and intellectual property (please explain)

- No, I am not working with 2 or more partners

- Yes, clear arrangements have been made regarding data management and intellectual property through a consortium agreement.

1.8 I can give an estimate of the size of the data collection; specifically, the number of participants or subjects (“n=”) in the collection and its size in GB/TB

- Not yet (please explain)

- Yes (please specify) → We estimated the size of the data collection based on factors such as the number of patients, project duration, type of data (e.g., questionnaires, healthcare consumption, focus groups), and data from previous studies. For WP1, involving 1,000 patients, the estimated data size is approximately 3.5 KB, based on the APOLLO trial database, which had half the number of patients and fewer questionnaires (1.2 KB). For WP2, with 1,500 patients, the estimated size is 5.25 KB. WP3 will involve about 1 GB of audio recordings from 6 focus groups (160 MB each). The total estimated size of data collected across all work packages is approximately 1.00525 GB.

Guidance:
Use "n=" (number of participants or subjects) and the number of giga-/terabytes per participants (or for the entire collection) to estimate **the size of the data collection**. You will need this to planning for instance storage capacity and budget.

1.9 The following end products I will make available for further research and verification (please elaborate briefly)

- Biobank

- Documentation of the research process, including documentation of all participants → This includes a comprehensive explanation of how the research was conducted, such as methodologies and procedures (as outlined in the study protocol).

- Software

- Data documentation → This refers to detailed descriptions of the data sets, including data structure, variables, and any coding used, which helps others understand and use the data effectively.

- Syntaxes → The scripts used to process the data in SPSS or R statistical software. This allows for the replication of data analyses and ensure consistency in data handling.

- Raw data → The original, unprocessed data collected during the research, preserving the integrity of the initial findings.

- (Several versions of) processed data → The data sets that have been cleaned, transformed, or analyzed, showing different stages or versions of data processing. These processed data sets are shared under the same terms as the raw data.

- Other products (please specify)

- Audiovisual material/ Images → The information videos created for patients, providing guidance on when to contact the hospital if they experience any problems with the prosthesis. This material offers additional context and practical information related to the study.

Guidance:
Make a description of your data collection and what you plan to do with your data. This helps to define what is needed for datamanagement. Consider what type of research data you will use and create in your project. Also consider what researchers need to know about them when they would want to reuse your data. You may therefore need to include synthaxes, algorithms, software, etc.
The guidance theme 'Data description' shows examples for various scenarios of quantitative and qualitative research data. They may hep to define the data that you will deliver and what you need to plan to make them reusable. (

1.10 During the project, I will have access to sufficient storage capacity and sites, and a backup of my data will be available (please elaborate briefly).

- Yes, I will make use of an external provider's services for storage and backup of my data: Castor Electronic Data Capture.

- Yes, I will make use of my institution's standard facilities for storage and backup of my data → To ensure the quality, safety, availability, and accessibility of research data, digital data will be centrally stored within the SharePoint environment at OLVG for WP1, and within the SharePoint environment at Tergooi MC for WP3. For WP2, data will be stored on the network drive of RHOC, which also includes automatic backups. Access to data is managed on a per-study-folder basis, ensuring that only members of the study team can access the data. Additionally, automatic version control is in place, allowing reversion to previous versions if needed. Paper study data and documents will be collected and stored in locked cabinets at the respective centers responsible for each WP: OLVG for WP1, Tergooi MC for WP3, and RHOC for WP2. These cabinets are accessible only to the study team. To ensure that research data cannot be traced back to individual patients, consent forms and the key list (subject identification code list) will be stored separately from the study data.

- Yes, I will personally facilitate the storage and backup of my data

- No, I have not yet secured a storage location (please explain)

Guidance:
Please consider the following when it comes to data storage:

- The properly organised **storage** and **backup** of data is necessary to prevent data from becoming lost due to technical problems or human error.

- Seek advice on **your research institute's storage facilities**. Contact your IT department, SURF, or any external service provider your organisation uses.

- Storage on laptops, hard disks or external media is generally risky. It is preferable to use robust, properly managed storage facilities provided by **the institution's IT department**. Automatic backups by the IT department are also safer than manual backups.

- When using **external services** you must ensure that there is no conflict with the policy of the body/ies funding the research (e.g. accessibility of data) or with the policy of your department or institute (e.g. security of sensitive data).

If you **manage** the storage and backup of your research data **yourself**, always consider: the scale of and growth in your data set, storage locations and capacity, version management, backups and technical and organisational measures to secure the data.

2. LEGISLATION (INCLUDING PRIVACY)

2.1 I will be doing research involving human subjects, and I am aware of and compliant with laws and regulations concerning privacy sensitive data.

- No, I will not be doing research involving human subjects; proceed to section 3 (Making data findable)

- The Wet Medisch-Wetenschappelijk Onderzoek met Mensen (WMO, or Medical Research (Human Subjects) Act) does not apply to my project. → This is likely applicable to WP3, given the nature of the study design. We are currently in the process of having this assessed to ensure compliance with the right regulations.

- Yes, I will involve human subjects in my research. I will comply with the Algemene Verordening Gegevensbescherming (AVG) → This applies specifically to WP1 and WP2.

- The Wet Medisch-Wetenschappelijk Onderzoek met Mensen (WMO, or Medical Research (Human Subjects) Act) applies to my project; I will have it reviewed by a Medical Research Ethics Committee. In addition I will comply with the Kwaliteitsborging Mensgebonden Onderzoek (Quality Assurance for Research Involving Human Subjects) → This applies specifically to WP1 and WP2.

- Wet op de Geneeskundige Behandelingsovereenkomst (Medical Treatments Contracts Act)

- Gedragscode Goed gebruik van lichaamsmateriaal (Code of Conduct for Responsible Use of Human Tissue)

Guidance:
Background information may be found on Laws and regulations on data management and FAIR data.

2.2 I will be doing research involving human subjects, and I have (a form of) informed consent from the participants for collecting their data.

- Yes, and this informed consent allows for the reuse of data (note that in the Code of Conduct for Medical Research, 'reuse' is also referred to as 'further use')

- Yes (please describe the form this consent takes)

Guidance:
Background information may be found on Laws and regulations on data management and FAIR data.

2.3 I will be doing research involving human subjects, and I will protect my data against misuse.

- Yes, the data will be anonymized. I realize that this will limit the options for re-use of my data. (explain)

- Other (please explain)

- Yes, the data will be pseudonymised (please explain how this will be done, and by which organization) → Each participating center will create a record in Castor EDC for every new participant. This record will automatically be assigned a unique number. Each initiating center will have access to the record numbers corresponding to their respective work package (WP). The unique numbers for participating centers are structured with a letter followed by a number (e.g., for Deventer Ziekenhuis: D001, D002, etc.). The key that links the record number to the patient will always remain securely within the participating center and separately from the data

Guidance:
Background information maybe found on Laws and regulations on data management and FAIR data.
Note:

- In some cases data are considered to be too sensitive to share. Also anonymising / pseudonymising the data may not be sufficient to prevent them from being identifiable. Or it is just not feasible to do (as, e.g., in tape recorded or video interviews).

- In these cases however, it is still possible to **share metadata of your dataset**. In that way, you at least show that these data exist.

- Research data management and stewardship differs in some aspects for **quantitative and qualitative research**. It is ZonMw's intention to accommodate both types of research in this DMP template.

- In case you come across conflicting or missing items, please explain in the DMP or contact openscience@zonmw.nl

2.4 I will stick to the privacy regulations of my organization

- Yes

- My organization does not have any regulations in this field, so I will personally make sure that the measures taken to guarantee the privacy and non-traceability of data are upheld (explain)

3. MAKING DATA FINDABLE

3.1 The data collection of my project will be findable for subsequent research. E.g., on a catalogue, a web portal, or through the search enginge of the repository (note: this is **key item 3**, which you should report to ZonMw at the end of your project).

- Yes, it can be found through the search engine of the archive or repository in which it is stored (please specify)

- Yes, it can be found through an online (metadata) catalogue or web portal (please specify)

- No, I have not yet chosen an archive or catalogue/web portal (please specify) → We are still evaluating options for making the data collection findable for subsequent research. Our goal is to ensure a consistent approach across WP1, WP2, and WP3. We are considering several repositories, including Dataverse NL (provided that a UMC member is part of the project group), 4TU.ResearchData as an international data repository, and potential solutions available within LUMC, where our project leader is also affiliated as a professor. We will finalize the repository selection as the project progresses.

Guidance:
Key item 3: The online catalogue or web portal where the dataset is registered
In addition to the persistent identifier, the findability of you dataset will be enhanced by registering the dataset in an online catalogue or web portal with a search engine. In this way, the dataset is findable for other potential users.
You can register the dataset on such a catalogue. This does not mean however that the data themselves are stored there. Rather, you register information about your dataset (metadata), and provide a reference through a persistent identifier.
The information about the dataset may include title, description, research goal and contact information and conditions for getting access to the data (**key item 4**), etc. It may be generic information, or specific information aimed at a research community.
An example of an international catalogue of databases can be found on FAIRsharing.org.
If possible, you use a catalogue that is common in your field of research. A good example of a specialised catalogue is zorggegevens.nl. This online catalogue lists a large number of datasets/databases from health research, along with descriptive metadata.
More examples are listed in the guidance theme **Overview of platforms, catalogues and repositories** for searching and reusing databases and collections.
It is important to register your dataset in a catalogue. In that way other researchers know that certain data already exist. They can decide to contact the data producer to agree on reusing the data in new research, instead of producing similar data again. Also if it is not possible to share your dataset, for instance because of privacy-sensitive data or because of intellectual property, it is still important to show that the data are already there.
Note: if you store your data in a repository (**key item 2**) that also has a search engine and functions as an online catalogue, it is not necessary to register your dataset in another searchable resource.
You are required to provide ZonMw with link of the searchable resource in your dataset is registered, at the end of your project.

3.2 I will use a metadata scheme for the description of my data collection (note: this is **key item 7**, which you should report to ZonMw at the end of your project).

- Yes, The metadata scheme includes metadata elements about the way my data are collected, preserved, etc (provenance) (please specify)

- No, I have not yet chosen a metadata scheme (please specify)

- Yes, I will use a metadata scheme specific for my field of research (please specify)

- Yes, I will use a generic metadata scheme (please specify) → We have selected the Dublin Core metadata schema due to its simplicity and effectiveness. Its 15 basic elements are straightforward and will comprehensively cover the essential details of our data.

Guidance:
Key item 7: Metadata scheme (preferably specific for your research discipline)
To make your dataset reusable, you should use a metadata scheme to describe your data. It is advised that you start with this fairly early in your project. After the project it is generally a lot harder and more work to do. There are already multiple standards available for different fields of research:
FAIRsharing.org provides an extensive catalogue of standards used in biomedical research.
When you register your dataset in an online catalogue (**key item 3**) you are in fact already asked to provide a set of metadata. The metadata asked for an online catalogue are mainly focused on improving the findability and accessibility of your data. Also the ZonMw key items listed in this document, are an example of metadata that describe certain features of your dataset.
However, the metadata in schemes like those listed on FAIRsharing.org are far more detailed and domain specific. These specific metadata schemes are aimed at improving the interoperability and reusability of the data.
Finally, if there is no metadata scheme available yet for your research discipline, you can use a generic metadata scheme, like Dublin Core.
More detailed information on metadata, see the **guidance on Metadata** on this site.
You are required to provide ZonMw with a link of the metadata scheme(s) that (you aim to) use with your dataset.

3.3 I will be using a persistent identifier as a permanent link to my data collection (note: this is **key item 1,** which you should report to ZonMw at the end of your project).

- No, I will not be using a persistent identifier (please explain)

- Yes, I will be using a persistent identifier (for instance a DOI code). Please specify which type of persistent identifiers you will use → The type of persistent identifier we will use is a DOI (Digital Object Identifier). This will serve as a permanent link to our data collection, providing a stable and consistent reference that ensures reliable access to the metadata and citation over time.

Guidance:
Key item 1: The Persistent Identifier referring to the dataset
A Persistent Identifier (PI) is an online permanent referral to a digital object that is independent of its storage location. The digital object in this case is the dataset itself, or metadata that describe what the dataset is about (see key item 7). This PI is a unique 'label' (usually in the form of a code) and is created by a (certified) data archive or repository. With a PI the dataset, or a description of it, can always be found on the internet, even when the name or location of it is changed since its creation. They are essential for ensuring Findability and sustainable archiving of your dataset. In addition, a PI enables you to cite your data in publications. Examples of PI's are DOI, Handle, URN of ARK.
For more information on PIs, see the web pages of the International DOI Foundation (IDF), Datacite (TUDelft). The course of RDNL is also a good option to learn more about the storing, managing, archiving and sharing of data.
With this section of your DMP, you will generate information about **key item 1:** A persistent identifier (PID) (e.g., a DOI-code) that refers to the dataset *(provide the code)*
This item also corresponds to FAIRmetric F1: (meta)data are assigned a globally unique and persistent identifier

4. MAKING DATA ACCESSIBLE

4.1 Once the project has ended, my data will be accessible for further research and verification.

- No (please explain)

- Yes, after an embargo period (please explain)
- Yes, immediately, but only upon request and if approved by the project group.

Guidance:
EMBARGO?
It could be that an embargo period needs to be observed, during which your data will not be accessible, for example for publication, intellectual property belonging to certain companies or commercial interests associated with exploitation of the research results.
ZonMw's grant terms and conditions stipulate a 3-month embargo, and a maximum of 9 months for patents.
Explain the reasons for the embargo and how long it will last.
If necessary, ZonMw will help you determine the duration of the embargo.
For more information see:

- Information on cofinancing on the ZonMw website.
- Laws and regulations concerning ZonMw

FAIR data =/= OPEN data
Note that there is a difference between open data and FAIR data.
Complying with the FAIR principle helps to make your data reusable. However, FAIR data are not necessarily OPEN data.
With the 'A' in FAIR, you can set conditions for sharing your data.

On the other hand, OPEN data are not necessarily reusable. They need to be FAIR to be able to reuse them.

4.2 Once the project has ended, my data collection will be publicly accessible, without any restrictions (open access).

- Yes, please proceed immediately to section 5 (Making data interoperable)
- No, there will be access restrictions to my data collection (please explain) → All data access requests will be discussed with the entire project group. Following this, the requests will be evaluated to ensure they comply with the CC-BY-NC-SA (Attribution-NonCommercial-ShareAlike) license for data sharing. Access will be subject to several factors, including collaboration agreements, data security, participant approval for further research, and any associated costs.

4.3 I have a set of terms of use available to me, which I will use to define the requirements of access to my data collection once the project has ended (please provide a link or persistent identifier; also note that this is a **key item 4**, which you should report to ZonMw at the conclusion of your project).

- Not yet, the terms of use will be determined by my institution to ensure they remain applicable to the prevailing laws and regulations at the time of the request.
- Yes, my institution employs internationally available terms of use
- Yes, my institution has drafted a set of terms of use with the help of a legal advisor

Guidance:
Key item 4: the terms of use for other researchers to use the dataset
ZonMw aims at reusable data according to its grant requirements. Therefore, ZonMw promotes researchers to create FAIR data. FAIR data, however, are not necessarily OPEN for anybody. With the 'A' of accessible in FAIR, you as a researcher can state the conditions by which the data will be shared.
If the reuse of your dataset is bound to specific conditions, or in other words there is *restricted access* to your dataset, other researchers must be able to view the terms of use. These must be findable online, e.g. through the website of your institute, or the catalogue or repository (key items 2 and 3).
The terms of use have to be made available by your institute or research group and should not be personal. Other researchers should be able to find out who they need to contact if they want to make use of the data.
The legal status of the licenses and conditions for reusing the data have to be clear. You can use international standards for the terms of use, or you can formulate them yourself together with a legal advisor.
Some of the criteria you can think of are:

- Collaboration in using the dataset, including agreements on publication and authorship
- The sharing of data for commercial purposes, taking into account the provisions of state aid law
- Conditions related to data security
- The approval of the participants allows for further research using this dataset, including linkage (privacy, informed consent)
- Agreements on methodology
- The way in which the data is made available
- The period of access
- Reimbursement for costs, e.g. in obtaining the data
- A committee decides whether access is granted

4.4 In the terms of use restricting access to my data, I have included at least the following:

- The permitted period of use of the data set
- The manner in which the data set can be accessed
- The sharing of data for commercial purposes, taking into account the provisions of state aid law
- The approval of the participants allows for further research using this data set
- Agreements on methodology
- Whether or not the data set may be linked with another data set (for reasons of privacy)
- Collaboration in using the data set, including agreements on publication and authorship
- Conditions related to data security
- Other (please explain)
- The reimbursement of costs, for example in obtaining the data
- A steering committee, programme committee or project leader will be charged with approving data requests

Guidance:
See guidance on question 4.3

5. MAKING DATA INTEROPERABLE

5.1 I will select a data format, which will allow other researchers and their computers (machine actionable) to read my data collection (note: this is **key item 5**, which you should report to ZonMw at the end of your project).

- Yes (please specify) → We will use the preferred formats recommended by DANS (https://dans.knaw.nl/nl/bestandsformaten/), our data is accessible and interoperable for other researchers. These formats are well-documented, widely supported, and suitable for our project.
- No (please explain)

Guidance:
Key item 5: The data format (preferably machine readable) of the dataset
To ensure long-term usability, accessibility and preservation of data, ZonMw recommends you to use a 'preferred' file format. DANS has made an extensive list of preferred formats. DANS is confident that preferred formats will offer the best long-term guarantees in terms of usability, accessibility and sustainability.
As a general guideline, DANS believes that the file formats best suited for long-term sustainability and accessibility:

- Are frequently used
- Have open specifications
- Are independent of specific software, developers or vendors

In practice, it is not always possible to use formats which satisfy all of these criteria.
Non-preferred formats are file formats that are widely used in addition to the preferred formats, and which will be moderately to reasonably usable, accessible and robust in the long term. DANS favours the use of preferred formats and recommends depositors to try to deposit data as much as possible in preferred formats.
ZonMw follows the recommendations of DANS for datasets from ZonMw's projects.
You are required to provide ZonMw with a link to (or name of) the data format dataset at the end of your project.

5.2 I will select a terminology for recording my data (e.g., code, classification, ontology) that allows my dataset to be linked or integrated with other datasets (note: this is **key item 6**, which you should report to ZonMw at the conclusion of your project).

- No (please explain)
- Yes, metadata standard (please specify) → We will adhere to the terminology, coding, and classification standards commonly used in orthopedic scientific research. We have reviewed the data dictionaries of the LROI and NOV and will use these standards as much as possible to ensure consistency and accuracy.

Guidance:
Key item 6: The terminology (e.g., code, classification, ontology) for the data
For your data to be interoperable, understandable and usable for humans, but more importantly also for computers, you have to 'speak a common language'. For instance, we agree to speak English or Dutch. Another example is that we agree to register weight in kilograms. Moreover, an interoperable datasets can be linked or integrated with other datasets.
For your research data this means: use a terminology for recording your data (codes, classifications, ontologies) that are commonly used in your field of research.
This key item relates to a complex part of your DMP. **Make good arrangements for this aspect of your study from the start of the project.** It is much more work to do it in retrospect!
For clinical research, you will find more information at Nictiz **('third layer of interoperability'), and** CASTOR (with tools for creating reusable data in clinical research, including the various layers of metadata, and coding metadata). A commonly used terminology in health care is SNOMED CT.
For biomedical research you can find more about ontologies on Bioportal.
For qualitative research you can find more information about software and interoperability of qualitative data in the guidance about Qualitative research data. There are specific metadata schemes for **qualitative data collections**:

- The Data Documentation Initiative Alliance is for the documentation of, among other things, social science and health research.
- For designing a metadata scheme that fits your research community and type of qualitative data, it may be helpful to look into the framework for describing and reusing metadata blueprints: the Component MetaData Infrastructure (CMDI), initiated by CLARIN. The "components" are building blocks for description of your data. They can include field definitions, and they can be grouped into a ready-made description format (a "profile"). Both are stored and shared with other users in the Component Registry to promote reuse.

Within a single research project, multiple standards for data terminology can be used. For example, standards used in a small subfield of a scientific discipline, standards derived from a specific protocol or standards required by a renowned register for data archiving.
The most important thing is that you try to use – as much as possible - terminology standards etc. that are commonly used in your specific field of research, type of experiment, protocol and so on.

You are required to provide ZonMw with a link of the terminology standard(s) you use in your project, **preferably linking to a document or webpage describing the standards.**

5.3 I will be doing research involving human subjects, and I have taken into account the reuse of data and the potential combination with other data sets when taking privacy protection measurements.

- Yes → Participants will be given the option on the consent form to separately indicate whether their data can be used for additional research questions and whether they consent to being contacted for follow-up research. Additionally, the data will be pseudonymised to protect participants' privacy.

- No (please explain)

Guidance:
Here ZonMw wishes to establish whether you have arranged privacy protection in such a way that your data cannot in principle be linked with other data sets.
In **research involving human subjects** certain ethical and legal requirements pertain to the linking of data. If the person has given their express **permission** for the secondary use of their data (including linking it with other data sets), the **identifying code** may be their BSN (Dutch tax and social security number), date of birth or postcode. If no permission has been given, the code must be a **pseudonym** that cannot be traced to the individual.
If the data are **anonymised** no permission is required, but it is not possible to link the data.
A trusted third party such as ZorgTTP can provide support with the exchange and accessibility of data sets containing privacy-sensitive information

6. MAKING DATA REUSABLE

6.1 I will ensure that the data and their documentation will be of sufficient quality to allow other researchers to interpret and reuse them (in a replication package).

- I will perform quality checks on the data to ensure that they are complete, correct and consistent (please explain) → We will perform quality checks by conducting random sample audits and continuous monitoring throughout the data collection process to ensure that the data is accurate, complete, and consistent.

- I will document the research process (please explain) → We will document the research process through progress reports, which will be submitted to ZonMw as part of our project requirements.

- In addition, I will take further quality assurance measures (please specify) → We will implement additional quality assurance measures such as regular review meetings with the research team to address any emerging issues and ensure adherence to research protocols.

- I will document the software used in the course of the project (please specify) → We will document all software used during the project, including version numbers and supplementary materials.

Guidance:
For the reuse of data and possible replication of the research, you must archive at least the following **data and documentation**:

- the raw data (if you have reused existing data, you may have processed data rather than 'raw' data; in that case, the existing data must already be permanently archived, or must now be archived);

- the data on which publications are based;

- documentation on the research methodology used (such as code books, data manuals, metadata compilation, machine settings, use of SOPs, version management etc.), project proposal, approval from ethics committees such as METC, all stakeholders (researchers, laboratory assistants, test subjects etc.) in short, everything needed to 'retrace the steps'.

- When storing **qualitative data**, describe the procedure used to transcribe the data (including conventions and symbols).

Together, this is known as a '**replication package'**.

Details on this minimum set of information, focused specifically on research covered by the Medical Research (Human Subjects) Act (WMO), can be found in the publication "Quality Assurance for Research Involving Human Subjects 2.0" (NFU, 2012). Appendix 3 to this publication lists the documents that must be archived for the preparation, implementation and completion of this type of research.

6.2 I have a number of selection criteria, which will allow me to determine which part of the data should be preserved once the project has ended. (see also question 1.9 and 6.1)

- Yes → We will apply specific, local selection criteria to determine which data should be preserved after the project ends. In compliance with regulations, we will ensure that all research data is retained for at least 15 years.

- Some or all of the data must be destroyed once the project has ended, because of a contract or law

- No

Guidance:
You must archive research data for reuse or to verify and validate research results. Consider at least the replication package, as mentioned in 6.1. However, you do not need to preserve all data. In some situations it is easier or less costly to generate exactly the same data again than to store that same data. In some circumstances, it may also be in the interests of privacy to generate data again rather than storing it. To help you determine which research data constitute valuable source material for further research, DANS has drawn up a **checklist** setting out 'General guidelines for the selection of research data for storage'. The guidelines set out the main reasons for archiving research data in the long term. The checklist can be used by individual researchers, research groups, institutions, archive managers and funding bodies. The Research Data Netherlands course (RDNL) also includes a user-friendly **decision diagram for data selection**.

6.3 Once the project has ended and the data have been selected, I can make an estimate of the size of the data collection (in GB/TB) to be preserved for long-term storage or archival.

- Yes (please specify) → Once the project is complete and the data have been selected, we will be able to precisely determine the amount of storage required for each WP in each coordinating center.

- Not yet (please explain)

6.4 I will select an archive or repository for (certified) long-term archiving of my data collection once the project has ended. (note: this is a key item, which you should report to ZonMw at the conclusion of your project)

- Yes, and this archive has a data seal of approval (please specify the archive)

- Yes, and this archive has a different form of certification (please specify the archive and certification)

- Not yet

- Yes, and this archive meets certification criteria and intends to get certified (please explain how your data will remain accessible and reusable in the long term) → For digital data, WP1 and WP3 will use SharePoint, and WP2 will use a network drive, in line with our organization's data management practices to ensure long-term accessibility. For paper data, WP1 documents will be archived with Deudekom in Duivendrecht, Tergooi MC's paper data will be stored at their facility in Kampen, and RHOC's paper data will be kept in a locked cabinet in Zoetermeer. Each coordinating center follows its local archiving procedures, ensuring data remains accessible and reusable as required.

Guidance:
Key item 2: The digital repository where the dataset is archived
At the end of your project, you are required to archive your data sustainably in a data repository (also called a data archive). Preferably, this should be done in a certified repository (core trust seal), as is listed here
Examples of Dutch data repositories with background information about data archiving are DANS, SURF, and 4TU.Centre for research data.
If you are used to archive your data on international repositories, you can keep on doing so. Especially when this is required by co-funders or scientific papers. In that case you do not have to archive your data on a Dutch repository as

well. In case you decide to store your data in the local IT facility at your institution, be sure that the data set is curated, retrievable and accessible. In this case you are required to register your data in an online catalogue, or web portal as well (key item 3). Read more in the guidance themes **Data repositories and catalogues** and **Overview of platforms, catalogues and repositories** for searching and reusing databases and collections. Read more in the guidance for **Qualitative research data** about facilities for archiving. You are required to provide ZonMw with the link of the digital archive at the end of your project.

6.5 Once the project has ended, I will ensure that all data, software codes and research materials, published or unpublished, are managed and securely stored. Please specify the period of storage.

- No, specify the guidelines and the number of years (please explain)
- Yes, in accordance with VNSU guidelines (please specify the number of years)
- Yes, in accordance with other guidelines (please explain, and specify the guidelines and the number of years) → We will manage and securely store all data, software codes, and research materials in accordance with our institutional guidelines and applicable regulations. All data, software codes and materials will be preserved for at least 15 years, in compliance with standard practices and legal requirements for long-term storage (https://www.ccmo.nl/onderzoekers/bewaartermijnen-medisch-wetenschappelijk-onderzoek)

Guidance:
Different codes and guidelines specify different storage periods.
The Netherlands Code of Conduct for Research Integrity (2018) states: "Ensure that all data, software codes and research materials, published or unpublished, are managed and securely stored for the period appropriate to the discipline(s) and methodology concerned" (paragraph 4.4 (13)). The NFU project Data4LifeSciences mentions in its data stewardship guideline HANDS 2.0 specifies a period for medical research.

6.6 Data management costs during the project and preparations for archival can be included in the project budget. These costs are:

- Unknown (please explain)
- Amount (please elaborate) → *Not publicly disclosed; covered within the overall project budget.*

6.7 The costs of archiving the data set once the project has ended are covered.

- Yes (please elaborate) → *Archiving costs are covered within the allocated project budget (ZonMw funding).*
- Not yet (please explain)

Guidance:
When **estimating the costs** you must distinguish between:

(1) the costs of data management **during the project** and the preparation of data for archiving, and
(2) the costs of permanent storage and archiving **after** the project.

Ad(1) These costs can be included in the budget submitted as part of your grant application. The costs of data management refer in part to:

- the time spent on metadata compilation and documentation during the study;
- **the type of data** and the **size** of the collection (you will have made an estimate of this in *part 1. General features of the project*);
- storage and securing of data during the project.

Ad(2) The costs of long-term storage will be borne by the institutes. A good picture exists of the costs of this and how they break down. They are at any rate determined by the type of data and the size (number of subjects and giga-/terabytes of the **selected dataset** (*see selection process in questions 2 and 3*)).
