# Supplementary material for: Is check-up on demand non-inferior to routine follow-up at one year after total hip or knee arthroplasty in terms of clinical outcomes and cost-effectiveness? Protocol for a randomized stepped-wedge hybrid effectiveness de-implementation trial
Source: PLoS One. 2026 Mar 17;21(3):e0343627. doi: 10.1371/journal.pone.0343627 (PMC12994803; doi:10.1371/journal.pone.0343627)
Supplement: S8 File — (PDF) [file pone.0343627.s008.pdf]

Information for participating in a health care evaluation

Routine follow-up appointment 1 year after a hip or knee replacement, necessary or not?

Official English titel: Routine follow-up 1 year after Hip-And Knee Arthroplasty (HAKA): wasting resources or appropriate healthcare?

Introduction

Dear Sir/Madam,

With this information letter, we would like to ask if you are willing to take part in a study. Participation is voluntary. You are receiving this letter because you will soon be receiving a hip replacement ("new hip"/"artificial hip") or a knee replacement ("new knee"/"artificial knee"). This letter explains the study and what we are asking of you. It contains a lot of information. Please take the time to read it carefully and decide whether you would like to participate. If you do, you can fill out the form in **Appendix C**.

Why this study?

After receiving a hip or knee replacement, patients normally receive follow-up check-ups. These usually include an X-ray and an appointment with the doctor. When and how often these check-ups take place can vary from hospital to hospital. At some hospitals, patients are scheduled for a standard check-up **1 year after surgery**. At other hospitals, this check-up is not standard practice. With this study, we want to investigate:

- Whether it is safe to stop doing the 1-year check-up;
- Whether skipping this check-up affects your recovery or overall well-being;
- What the costs are for the patient, the hospital, and society.

The answers will help us make better decisions in the future about when and how often patients should receive follow-up care.

What do we ask of you?

If you take part in this study, there are **two possible options**:

1. You **do** come to the hospital for a standard check-up **1 year after surgery**,
   or
2. You **do not** come to the hospital for a standard 1-year check-up, but you **can request one at any time** if you feel the need.

Whether or not you receive the standard check-up depends on the timing of your surgery. We will also ask you to fill out 5 questionnaires.

Ask your questions

You can make your decision based on the information in this letter. You may also ask your treating physician any questions you have. Or talk it over with your partner, family, or friends. If you wish, you may also contact an independent expert for more information (see **Appendix A** for contact details). You can also visit https://www.zorgevaluatienederland.nl or watch the information video at https://www.zorgevaluatienederland.nl/about.

1. General information

This study is organized by OLVG, in collaboration with Tergooi MC, the Reinier Haga Orthopedic Center (RHOC), and the Dutch Orthopedic Association (NOV), and is being conducted in various hospitals and clinics across the Netherlands.

Participants in a medical research study are often referred to as subjects. Both patients and healthy individuals can be subjects. A total of 1000 subjects will participate (500 patients with a hip prosthesis and 500 patients with a knee prosthesis). The Medical Ethical Review Committee (METC) Leiden Den Haag Delft has approved this study.

Health care evaluation

This study is a health care evaluation. For many conditions, there are several possible forms of follow-up care. However, we do not always know which type of follow-up care is best for the patient. That's why we are conducting research on this, which is called care evaluation research (hereafter: care evaluation). In a care evaluation, we compare existing forms of follow-up care. We examine which care is more effective, less burdensome, and has the lowest costs. This allows us to better choose the best care for each patient. Since you will receive a standard form of care, there is no additional risk involved in participating in a care evaluation. By participating, you are helping to improve care for future patients.

2. What is the background of the health care evaluation?

Each year, approximately 36,000 patients in the Netherlands receive a hip prosthesis and 26,000 patients receive a knee prosthesis due to osteoarthritis. Within three months after surgery, the hip or knee prosthesis is checked. X-rays are taken and there is an appointment with a doctor or physician assistant (a medical care provider who works under the supervision of a doctor). This check-up is important for both the doctor and the patient. The frequency of follow-up visits after this check-up varies by hospital. Sometimes this happens every year, but sometimes only once every five years. We don't know whether a check-up one year after surgery is necessary. It might not be needed for patients without complaints to visit the hospital. This would save time for patients and healthcare workers, and prevent unnecessary X-ray exposure.

3. What is the purpose of the health care evaluation?

We want to investigate whether we can stop the check-up one year after surgery. The check-up at one year post-surgery would only be for patients who have complaints or concerns, for those referred by their general practitioner, or for patients who have been asked by their doctor to come in. We are also examining whether stopping the check-ups has an impact on the quality of life, and what patients think about these check-ups.

4. How does the health care evaluation proceed?

Hoe long does the health care evaluation last?

Your participation will last approximately 24 months in total.

Step 1: Can you participate?

First, we need to determine if you are eligible to participate. Therefore, the doctor or researcher will review your medical history.

Step 2: Which follow-up will you receive?

In this health care evaluation, we will create two groups:

- **Group 1.** People in this group will receive 2 follow-ups: one within 3 months and another 1 year after the surgery. For both follow-ups, they will have an appointment with a doctor or physician assistant and will have an X-ray taken. They can also contact the medical team if they have any complaints or concerns.
- **Group 2.** People in this group will receive only one follow-up within 3 months. For this follow-up, they will have an appointment with a doctor or physician assistant and will have an X-ray taken. They will not receive a standard follow-up 1 year after the surgery. They will be given clear instructions on how and when to contact their general practitioner or the hospital, such as if they have complaints or concerns.

Additionally, in both groups, the general practitioner or specialist can always request an additional appointment if needed.

Which group you are in depends on the timing of your surgery.

Each hospital begins with follow-ups as described for group 1. After a certain period, the hospital switches to the follow-ups as described for group 2. The time at which your hospital makes this transition will be determined by random selection. This means that the group you are assigned to depends on when you have your surgery and what follow-up your hospital is conducting at that time.

Step 3: Research and measurements (see Appendix B: Overview of measurements)

- You will visit the hospital 1 or 2 times. Each visit lasts about 30 minutes. You will have an X-ray and an appointment with a healthcare provider.

You will receive 5 questionnaires from us:

- Before the surgery
- One year after the surgery
- 15 months after the surgery
- 18 months after the surgery
- Two years after the surgery

Completing the questionnaires will take about 30 minutes each time. You can fill them out at your own pace. You can also receive phone assistance from a researcher if needed. The questions will cover your health, pain in your hip or knee, how well you can move your hip or knee, your satisfaction with the surgery, and any costs you have incurred. Additionally, we will ask about your background, such as your ethnicity, employment, and education level. This will help us understand which people may benefit more or less from a follow-up 1 year after the surgery. The questionnaire was created in collaboration with patients, so it is easy to understand and does not ask too much from you. The questionnaire will be sent by email. If you prefer to receive it by mail, that is also possible.

- About 1 year after your surgery, you can participate in a group discussion about your experience with the follow-ups. You can choose whether or not to participate. In Appendix C, you can indicate whether we may share your contact information with Tergooi MC. They may then contact you for more information.

What is different from regular care?

There is not much different in this health care evaluation compared to regular care. In regular care, whether or not you receive a follow-up 1 year after surgery depends on the hospital or the specific doctor within the hospital. The only difference is that you will be filling out questionnaires. These questionnaires help us determine whether a standard follow-up 1 year after surgery is useful.

5. What agreements do we make with you?

We want the health care evaluation to proceed smoothly. Therefore, we make the following agreements with you:

- You will not participate in another medical research study during this health care evaluation if it affects the current study.
- You will fill out all the questionnaires.
- You will contact the researcher if:
  - You are admitted to or treated in a hospital.
  - You experience any sudden health problems.
  - You no longer wish to participate in the health care evaluation.
  - Your phone number, address, or email address changes.

6. What negative effects or discomforts might you experience?

There are no discomforts or risks that you will experience.

7. What are the benefits and drawbacks of participating in the health care evaluation?

Participating in the health care evaluation will not provide you with any additional medical benefits. By participating, you are helping future patients.

Participating in the health care evaluation may also have some drawbacks. Here's a list of them. Please consider them carefully and discuss them with others:

- Filling out the questionnaires will take some time.
- If you are assigned to group 1, you may have an additional appointment with an X-ray, which you would not normally have at your hospital.
- During the extra appointment, an X-ray will be taken. You will be exposed to about 0.13 mSv of radiation. For comparison, everyone in the Netherlands receives about 2.5 mSv of radiation annually from natural sources.
- The costs for the hospital visit will be covered by your health insurance. This will count toward your deductible unless it has already been used. If you do not want to attend the appointment for this reason, you can still participate in the health care evaluation by only filling out the questionnaires.

Do you not want to participate?

You decide whether or not you want to participate in the health care evaluation. If you do not want to participate, it will not affect the care you receive. You will receive the follow-ups you would normally get after your surgery, as the hospital would typically do.

8. When does the health care evaluation end?

The researcher will inform you if there is any new information about the health care evaluation that is important to you. The researcher will then ask if you would like to continue participating.

In these situations, the health care evaluation will end for you:

- All research according to the schedule (see Appendix B) has been completed.
- You wish to stop participating in the health care evaluation. You can stop at any time. Please inform the researcher immediately. You do not need to explain why you are stopping. You will then receive the follow-ups you would normally receive after your surgery.
- One of the following entities decides that the health care evaluation must stop:
  - o The sponsor (OLVG),
  - o The government,
  - o The funding body, or
  - o The medical ethical committee that evaluates the health care evaluation.

What happens if you stop participating in the health care evaluation?

The researchers will use the data collected up until the moment you stop.

9. What happens after the health care evaluation?

Will you receive the results of the health care evaluation?

About one year after the last participant has completed the health care evaluation, you will receive the main results of the health care evaluation by email or post from the researcher.

10. What do we do with your data?

If you participate in the health care evaluation, you also give permission for your data to be collected, used, shared, and stored.

What data do we store?

We store the following data:

- Your name
- Your gender
- Your (mailing) address
- Your phone number
- Your date of birth
- Health information about you
- (Medical) data we collect during the health care evaluation

Why do we collect, use, and store your data?

We collect, use, share, and store your data to answer the questions of this health care evaluation and to publish the results. We also need this data to apply the findings of the health care evaluation in practice.

How do we protect your privacy?

To protect your privacy, we assign a code to your data. We will only use this code on all your data. The key to the code is stored in a secure location at the hospital. When processing your data, we will always use only the code. In reports and publications about the health care evaluation, it will not be possible to trace the information back to you.

Who can view your data?

Certain individuals may view your name and other personal information without the code. These are people who check whether the researchers are conducting the health care evaluation correctly and reliably. The following people can access your data:

• A monitor working for the researcher

• National authorities, such as the Health and Youth Care Inspectorate

These individuals are required to keep your data confidential. We will ask for your consent for this

access. The Health and Youth Care Inspectorate can access your data without your consent.

Who will receive your contact details?

The research team at your hospital can view your contact details. We will ask your consent to share your phone number and (email) address with the research team at OLVG. They will use your phone number to contact you if you have not (fully) completed a questionnaire. We will use your (email) address to send you the key outcomes of the health care evaluation.

How long will we store your data?

Your data will be stored for 15 years, both at the hospital and with the sponsor (OLVG).

Can we use your data for other research?

Your data may also be important for other scientific research about hip and knee prostheses after the health care evaluation ends. We will store your data for 15 years at the hospital for this purpose. In the consent form, you can indicate whether you agree to this. If you do not consent, you can still participate in this health care evaluation.

Can you withdraw your consent for the use of your data?

You can withdraw your consent for the use of your data at any time. Please inform the researcher if you wish to do so. This applies to both the use in this health care evaluation and in other research. If you withdraw your consent, researchers can still use the data collected before the withdrawal.

Do you want to know more about your privacy?

- If you want to know more about your rights regarding the processing of personal data, please visit www.autoriteitpersoonsgegevens.nl.
- If you have questions about your rights or a complaint about the processing of your personal data, please contact the person responsible for processing your personal data. For your health care evaluation, that is:
  o [Institution name] See Appendix A for contact details and website.
- If you have complaints about the processing of your personal data, we recommend discussing them first with the research team. You can also contact the Data Protection Officer at [the institution], or file a complaint with the Dutch Data Protection Authority.

Where can you find more information about the health care evaluation?

You can find more information about the health care evaluation on the following website(s): https://zorgevaluatienederland.nl/evaluations/haka-1-year-fu and https://onderzoekmetmensen.nl. After the health care evaluation, the website may display a summary of the results. You can find the evaluation by searching for “Standard follow-up 1 year after a total hip or knee prosthesis: wasted effort or appropriate care? (HAKA study - 1 year follow-up)” (number: NL-009246).

11. Will you be compensated for participating in the health care evaluation?

You will not receive compensation for participating in this health care evaluation. The standard follow-ups, including the 1-year post-surgery check-up, at the hospital are part of regular care. The costs are covered by your health insurance and may count toward your deductible.

12. Are you insured during the health care evaluation?

You are not additionally insured for this health care evaluation. Since participating in this health care evaluation carries the same risks as regular care for your hip or knee prosthesis, the researcher does not need to arrange additional insurance with the METC Leiden Den Haag Delft.

13. Do you have questions?

If you have any questions about the health care evaluation, you can contact the research team. If you would like advice from someone without a vested interest, you can contact Mr. van de Groes. He is knowledgeable about the health care evaluation but is not involved in conducting it. His contact details are provided in Appendix A. If you have a complaint about the health care evaluation, you can discuss it with the researcher or the doctor treating you. If you prefer not to do that, you can contact the complaints officer at your hospital. You can find their contact details in Appendix A.

14. How do you give consent for the health care evaluation?

You can first take your time to think about this health care evaluation. Then, you will tell the doctor or researcher if you understand the information and whether or not you want to participate. If you want to participate, you will fill out the consent form that is attached to this information letter. If you received the form on paper, please sign both consent forms and return them in the envelope provided. You will receive a fully signed version back. If you complete the form digitally, the researcher will send you a copy via email.

Thank you for your time.

[Name of the principal investigator at the participating center + name of the center]

On behalf of,

The HAKA research team

---

15. Appendices to this information

A. Contact Information

B. Overview of measurements

C. Consent Form

Appendix A: Contact Information for [name of the participating center]

[Researcher]: [for the principal investigator of the center: name, contact details, and availability]

[Research nurse/research doctor/nurse specialist]:

Independent expert: Mr. van de Groes

Complaints: [service or person with contact details and availability]

Data Protection Officer of the institution:

For more information about your rights: [Contact details [including website] of the responsible party(ies) for the processing of personal data]:

Appendix B: Overview of measurements

	Pre-operation	Surgery	Na de operatie				
			Within 3 months	1 year	1 year and 3 months	1 year and 6 months	2 years
Medical data	X						
Hip or knee prosthesis surgery		X					
Appointment with healthcare provider	X		X	X**			
X-ray	X		X	X**			
Questionnaires	X			X*	X*	X*	X*

* Extra for research

** Depending on which group you belong to

Appendix C: Consent Form (patient version)

Belonging to

Routine follow-up appointment 1 year after a hip or knee replacement, necessary or not?

Official English titel: Routine follow-up 1 year after Hip-And Knee Arthroplasty (HAKA): wasting resources or appropriate healthcare?

- I have read the information letter. I was also able to ask questions. My questions were answered sufficiently. I had enough time to decide whether I want to participate.
- I understand that participation is voluntary. I also understand that I can decide to stop participating at any time. I do not have to give a reason for stopping.
- I give the researchers permission to collect and use my data. They will only use my data to answer the research questions of this study.
- I understand that for the purpose of monitoring the study, certain people may review all of my data. These people are mentioned in the information letter. I give them permission to review my data for this purpose.

I give permission for my data to be stored and used for other research, as described in the information letter.	Yes ☐	No☐
I give permission for my phone number and (email) address to be shared with the research team so they can contact me if needed, as described in the information letter.	Yes ☐	No☐
I give permission for my contact details to be shared with Tergooi MC, so they may contact me 1 year after surgery to invite me to a group discussion.	Yes ☐	No☐
I give permission to be contacted after this evaluation study to be asked about participating in a follow-up study.	Yes ☐	No☐

- I agree to take part in this evaluation study.

My name (participant):………………………………..
Signature: ……………………… Date : __ / __ / __

I declare that I have fully informed this participant about the mentioned evaluation study. If new information becomes available during the study that may influence the participant's consent, I will inform them in a timely manner.

Name of researcher (or representative):): ………………………………..
Signature: ……………………… Date : __ / __ / __

The participant will receive a full information letter and a signed consent form..

Appendix C: Consent Form (researcher version)

Belonging to

Routine follow-up appointment 1 year after a hip or knee replacement, necessary or not?

Official English titel: Routine follow-up 1 year after Hip-And Knee Arthroplasty (HAKA): wasting resources or appropriate healthcare?

- I have read the information letter. I was also able to ask questions. My questions were answered sufficiently. I had enough time to decide whether I want to participate.
- I understand that participation is voluntary. I also understand that I can decide to stop participating at any time. I do not have to give a reason for stopping.
- I give the researchers permission to collect and use my data. They will only use my data to answer the research questions of this study.
- I understand that for the purpose of monitoring the study, certain people may review all of my data. These people are mentioned in the information letter. I give them permission to review my data for this purpose.

I give permission for my data to be stored and used for other research, as described in the information letter.	Yes ☐	No☐
I give permission for my phone number and (email) address to be shared with the research team so they can contact me if needed, as described in the information letter.	Yes ☐	No☐
I give permission for my contact details to be shared with Tergooi MC, so they may contact me 1 year after surgery to invite me to a group discussion.	Yes ☐	No☐
I give permission to be contacted after this evaluation study to be asked about participating in a follow-up study.	Yes ☐	No☐

- I agree to take part in this evaluation study.

My name (participant):………………………………..

Signature: ……………………… Date : __ / __ / __

I declare that I have fully informed this participant about the mentioned evaluation study. If new information becomes available during the study that may influence the participant's consent, I will inform them in a timely manner.

Name of researcher (or representative):): ………………………………..

Signature: ……………………… Date : __ / __ / __

The participant will receive a full information letter and a signed consent form..
