# Supplementary material for: Is check-up on demand non-inferior to routine follow-up at one year after total hip or knee arthroplasty in terms of clinical outcomes and cost-effectiveness? Protocol for a randomized stepped-wedge hybrid effectiveness de-implementation trial
Source: PLoS One. 2026 Mar 17;21(3):e0343627. doi: 10.1371/journal.pone.0343627 (PMC12994803; doi:10.1371/journal.pone.0343627)
Supplement: S9 File — (PDF) [file pone.0343627.s009.pdf]

HAKA study – 1 year follow-up

Study number
To be completed by researcher

Dear sir/madam,

You agreed to participate in our study "Routine follow-up appointment after a hip or knee replacement: necessary or not after 1 year?". As previously explained by the researcher, we would like to ask you to complete a questionnaire. This questionnaire consists of several sections.

Completing the questionnaire will take approximately 25 minutes. Please read the instructions for each questionnaire carefully before answering.

It is important that you complete this questionnaire ***within 1 week*** and ensure that ***all questions*** are answered. Please also remember to fill in the ***back of the pages***. Once completed, you can return the questionnaire to us using the enclosed return envelope. A stamp is not required.

This questionnaire is part of the study, in which you receive a questionnaire before surgery, at one year after surgery, and then at 15, 18 months and 2 years after surgery.

If you have any further questions about this study, please contact one of the investigators. The contact information can be found in the participant information you received at the beginning of the study.

Best regards,

The HAKA research team

Version 1.0 date: 02-10-2025

Date of completion
To be completed by researcher

Study number
To be completed by researcher

Date on which you completed the questionnaire: ______________________

Cost Questionnaire

The following questions are about the healthcare costs you have incurred **due to your operated hip or knee**.

Instruction: Please answer all questions and complete the tables if your answer is 'Yes'. If you do not know exactly how often, how much, or how many, please give your best estimate

1. In the **past 6 months**, have you had contact with a general practitioner **because of (problems with) your artificial hip or artificial knee?**

   □ No

   □ Yes, please fill in the table below.
   Note: Entering multiple response options is possible

Type of contact	Number of visit(s)
□ Visit general practice/clinic	Number ....
□ Telephone contact	Number ....
□ Home visit	Number ....

2. In the **past 6 months**, have you visited a therapist **because of (problems with) your artificial hip or artificial knee**?

   □ No

   □ Yes, please fill in the table below.
   Note: Entering multiple response options is possible.

Type of therapist	Number of visit(s)
□ Physical therapist	Number ....
□ Occupational therapist	Number ....
□ Manual physical therapist	Number ....
□ Exercise therapist (e.g. Mensendieck/Cesar therapist)	Number ....
□ Psychologist	Number ....
□ Other, namely...	Number ....

Version 1.0 date: 02-10-2025

Date of completion
To be completed by researcher

3. In the **past 6 months**, have you visited an alternative healer **because of (problems with) your artificial hip or artificial knee**?

□ No

□ Yes, please fill in the table below.
Note: Entering multiple response options is possible

Alternative healer	Cost per visit	Number of visit(s)
□ Chiropractor	$	Number ....
□ Acupuncturist	$	Number ....
□ Homeopath	$	Number ....
□ Other, namely...	$	Number ....

4. In the **past 6 months**, have you visited the emergency department of a hospital **because of (problems) with your artificial hip or artificial knee**?

□ No

□ Yes, please fill in the table below.

Number of visit(s)	Number of times you have been taken by ambulance to the emergency department
Number	Number ....

5. In the **past 6 months**, have you visited the hospital or had an online or telephone consultation with the hospital **because of (problems with) your artificial hip or artificial knee**?

□ No

□ Yes, please fill in the table below.
Note: Entering multiple response options is possible

Type of visit	Number of visit(s)
□ Orthopedic outpatient clinic	Number ....
□ Telephone/online consultation	Number ....
□ Other, namely..........................	Number ....

6. In the **past 6 months**, have you had an X-ray or scan because of **(problems with) your artificial hip or artificial knee**?

□ No

□ Yes, please fill in the table below.
Note: Entering multiple response options is possible

Type of procedure	Number of survey(s)
□ X-ray<br><br>□ Scan<br><br>□ Other, namely..........................	Number ....<br><br>Number ....<br><br>Number ....

7. In the **past 6 months**, have you been admitted to a care facility for **(problems with) your artificial hip or artificial knee**?

□ No

□ Yes, please fill in the table below.
Note: Entering multiple admissions is possible

What kind of institution have you been admitted to?	How many days were you admitted? *Note: Have you been admitted more than once? Then add all the days together*	Did you (if applicable) stay in the Intensive Care Unit (ICU) during your admission?
□ Hospital<br><br>□ Rehabilitation institution<br><br>□ Nursing home<br><br>□ Otherwise, namely...................	.... Days<br><br>....... Days<br><br>....... Days<br><br>....... Days	□ No<br><br>□ Yes,.......(days)

8. In the **past 6 months**, have you used any medications for (**problems with) your artificial hip or artificial knee**? If you were taking medications before your surgery but are now taking them specifically for your artificial hip or artificial knee, fill in yes.

□ No

□ Yes, please fill in the table on the next page.
Note: Entering multiple response options is possible

Medicines	How many days have you used this medicine in the past 6 months?
□ Regular painkillers (e.g. ibuprofen, paracetamol)	.... days
□ Diclofenac	.... days
□ Stomach protectant (e.g., omeprazole, pantoprazole)	.... days
□ Opiates (e.g., oxycodone, codeine, tramadol, morphine)	.... days
□ Antidepressants (e.g., paroxetine, amytriptiline)	.... days
□ Other, namely..........................	.... days

9. In the **past 6 months**, have you used assistive devices for **(problems with) your artificial hip or artificial knee**? If you used assistive devices before your surgery but now have them in use specifically for your artificial hip or artificial knee, fill in yes.

□ No

□ Yes, please fill in the table below.
Note: Entering multiple response options is possible

Resources	How long have you used this tool in the past 6 months?
□ Rollator walker	.... weeks
□ Stools	.... weeks
□ Wheelchair	.... weeks

□ Shower chair	.... weeks
□ Bathroom support brackets	.... weeks
□ Shower bag to keep leg dry	.... weeks
□ Bedpan	.... weeks
□ Other, namely..........................	.... weeks

10. **In the past 6 months**, have you had any help at home (for example, domestic help/home care) **because you had problems with your artificial hip or artificial knee**? If you had help before your surgery but now have it specifically for your artificial hip or artificial knee, fill in yes.

□ No

□ Yes, please fill in the table below.
Note: Entering multiple response options is possible

**Type of help**	**Number of hours per week**	**Number of weeks**
☐ Home care	. ...hours/week	.... weeks
☐ Help from family, friends, volunteers	. ...hours/week	.... weeks
☐ Paid domestic help	. ...hours/week	.... weeks

11. During the **past 6 months**, have you been unable to perform unpaid work **because of (problems with) your artificial hip or artificial knee**?

□ No

□ Yes, please enter in the table on the next page the number of hours per week and number of weeks you were unable to perform this work
Note: Entering multiple response options is possible.

**Unpaid work**	**Number of hours per week**	**Number of weeks**
☐ chores in and around your home	. ...hours/week	.... weeks
☐ Volunteer	. ...hours/week	.... weeks
☐ Study/Education	. ...hours/week	.... weeks
☐ Other, namely.................	.... hours/week	.... weeks

12. **Do you have paid work?**

    □ No (End of questionnaire)

    □ Yes, please the following questions.

13. **How many hours per week do you work?**

    . .... hours per week

14. **Over how many days are these hours divided?**

    . ..... days

15. In the **past 6 months**, how many working days did you have to ask for time off or call in sick **because of (problems with) your artificial hip or artificial knee?**

    . ..... working days

16. On a scale of 0 to 10, how would you rate your **overall work performance** in the **past 6 months**, with score 0 corresponding to the worst possible performance (nothing comes out of your hands) and score 10 corresponding to the best possible performance in your work (you are as productive as normal)?

    Circle the score on the scale below:

0 1 2 3 4 5 6 7 8 9 10

Worst possible performance **Best possible performance**

Pain score (NRS)

With these questions, we want to get an understanding of how much pain you have experienced **in your hip/knee (surgical side)** **in the past week**.

Instruction: would you please check the box below that best describes you?

1. How much pain from your hip/knee (surgical side) have you experienced in the past week....

...at rest?

No pain ↓ — *Very severe pain* ↓

❑ 0 ❑ 1 ❑ 2 ❑ 3 ❑ 4 ❑ 5 ❑ 6 ❑ 7 ❑ 8 ❑ 9 ❑ 10

2 ... during weight-bearing activities?

no pain ↓ — *Very severe pain* ↓

❑ 0 ❑ 1 ❑ 2 ❑ 3 ❑ 4 ❑ 5 ❑ 6 ❑ 7 ❑ 8 ❑ 9 ❑ 10

Satisfaction (NRS).

With these questions, we want to get an understanding of your satisfaction with the outcome of your operated hip/knee.

Instructions: would you please check the box below that best describes you?

1. **How satisfied are you (in general) with the outcome of your hip/knee surgery?**

Very dissatisfied ↓ — *Very satisfied* ↓

❑ 0 ❑ 1 ❑ 2 ❑ 3 ❑ 4 ❑ 5 ❑ 6 ❑ 7 ❑ 8 ❑ 9 ❑ 10

Physical functioning (PROMIS)

The following questions are about your physical functioning in general, not just about your operated hip or knee.

Instruction: Please respond to each question or statement by marking one box per row.

	Without any difficulty	With a little difficulty	With some difficulty	With much difficulty	Unable to do
Are you able to do chores such as vacuuming or yard work?..........................	☐ 5	☐ 4	☐ 3	☐ 2	☐ 1
Are you able to get in and out of a car?	☐ 5	☐ 4	☐ 3	☐ 2	☐ 1
Are you able to go up and down stairs at a normal pace?	☐ 5	☐ 4	☐ 3	☐ 2	☐ 1
Are you able to run errands and shop?.......	☐ 5	☐ 4	☐ 3	☐ 2	☐ 1
Are you able to bend down and pick up clothing from the floor?..............................	☐ 5	☐ 4	☐ 3	☐ 2	☐ 1
Are you able to lift 10 pounds (5 kg) above your shoulder?	☐ 5	☐ 4	☐ 3	☐ 2	☐ 1
	**Not at all**	**Very little**	**Somewhat**	**Quite a lot**	**Cannot do**
Does your health now limit you in doing vigorous activities, such as running, lifting heavy objects, participating in strenuous sports?..	☐ 5	☐ 4	☐ 3	☐ 2	☐ 1
Does your health now limit you in bathing or dressing yourself?	☐ 5	☐ 4	☐ 3	☐ 2	☐ 1
Does your health now limit you in putting a trash bag outside?	☐ 5	☐ 4	☐ 3	☐ 2	☐ 1
Does your health now limit you in doing moderate activities, such as moving a table, pushing a vacuum cleaner, bowling, or playing golf?	☐ 5	☐ 4	☐ 3	☐ 2	☐ 1

PROMIS® Item Bank v2.0 – Physical Function – Short Form 10b

Quality of life (EQ-5D)

The following questions are about your quality of life.

Instruction: Under each heading, please check the ONE box that best describes your health TODAY. **It does** not matter if this is because of your hip or knee, or for another reason.

1. Mobility
☐ I have no problems walking
☐ I have slight problems walking
☐ I have moderate problems walking
☐ I have severe problems walking
☐ I am unable to walk

2. Self-care
☐ I have no problems washing or dressing myself
☐ I have slight problems washing or dressing myself
☐ I have moderate problems washing or dressing myself
☐ I have severe problems washing or dressing myself
☐ I am unable to wash or dress myself

3. Usual activities (e.g., work, study, housework, family or leisure activities)
☐ I have no problems doing my usual activities
☐ I have slight problems doing my usual activities
☐ I have moderate problems doing my usual activities
☐ I have severe problems doing my usual activities
☐ I am unable to do my usual activities

4. Pain/discomfort
☐ I have no pain or discomfort
☐ I have slight pain or discomfort
☐ I have moderate pain or discomfort
☐ I have severe pain or discomfort
☐ I have extreme pain or discomfort

5. Anxiety/depression
☐ I am not anxious or depressed
☐ I am slightly anxious or depressed
☐ I am moderately anxious or depressed
☐ I am severely anxious or depressed
☐ I am extremely anxious or depressed

We would like to know how good or bad your health is TODAY.

This scale is numbered from 0 to 100.

100 means the <u>best</u> health you can imagine.
0 means the <u>worst</u> health you can imagine.

It does not matter if this is because of your hip or knee, or for another reason.

Mark an X on the scale to indicate how your health is TODAY.

Now, please write the number you marked on the scale in the box below.

YOUR HEALTH TODAY =

The best health
you can imagine

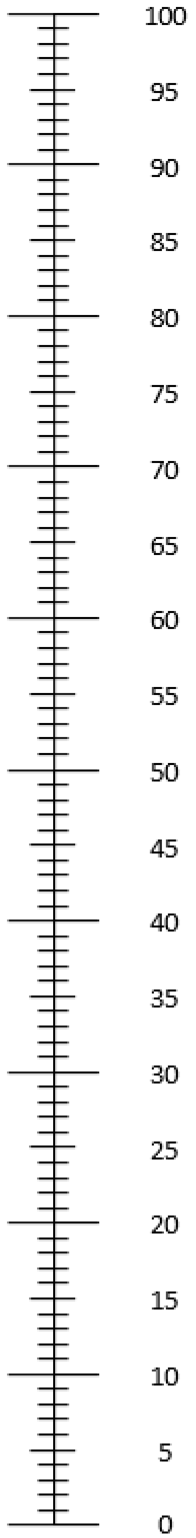

The worst health
you can imagine

© EuroQol Research Foundation. EQ-5D™ is a trade mark of the EuroQol Research Foundation. USA (English) v1.2

This is the end of the questionnaire.

We would appreciate it if you could take a moment to check that you haven't accidentally skipped any pages or questions.

Please return the completed questionnaire to us as soon as possible in the enclosed envelope. No stamp required.

We thank you very much for your cooperation!
